# Removing the Barrier in O–O Bond Formation Via the Combination of Intramolecular Radical Coupling and the Oxide Relay Mechanism

**DOI:** 10.1021/acs.jpca.4c00404

**Published:** 2024-05-06

**Authors:** Juan Angel de Gracia Triviño, Mårten S. G. Ahlquist

**Affiliations:** † Division of Theoretical Chemistry and Biology, Department of Chemistry, School of Engineering Sciences in Chemistry, Biotechnology and Health, KTH Royal Institute of Technology, 10691 Stockholm, Sweden; ‡ PDC Center for High-Performance Computing, School of Electrical Engineering and Computer Science, KTH Royal Institute of Technology, 10691 Stockholm, Sweden

## Abstract

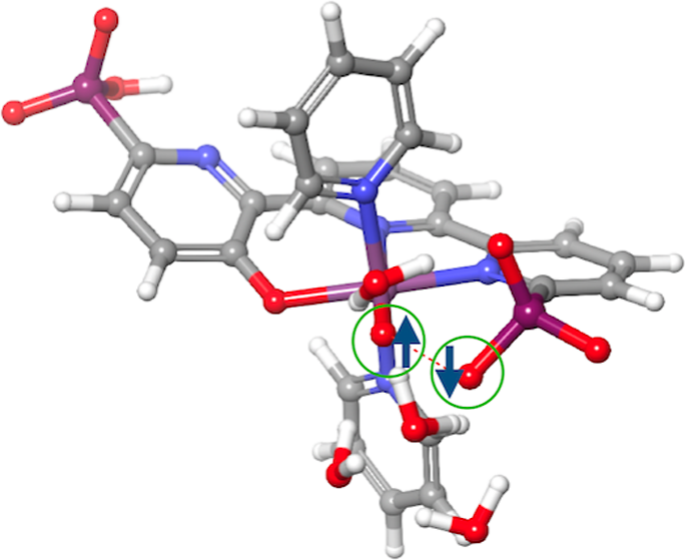

The Ru(tda) catalyst has been a major milestone in the development of molecular water oxidation catalysts due to its outstanding performance at neutral pH. The role of the noncoordinating carboxylate group is to act as a nucleophile, donating an oxygen atom to the oxo group, thereby acting as an oxide relay (OR) mechanism for O–O bond formation. A substitution of the carboxylates for phosphonate groups has been proposed, resulting in the Ru(tPaO) catalyst, which has shown even more efficient performance in experimental characterization. In this study, we explore the feasibility of the OR mechanism in the newly reported Ru(tPaO) molecular catalyst. We investigated the catalytic cycle using density functional theory and identified a variation of the OR mechanism that involves radical oxygen atoms in O–O bond formation. We have also determined that the subsequent hydroxide nucleophilic attack is the sole rate-limiting step in the catalytic cycle. All activation free energies are very low, with a free-energy barrier of 2.1 kcal/mol for O–O bond formation and 4.2 kcal/mol for OH^–^ nucleophilic attack.

## Introduction

**Figure 1:**
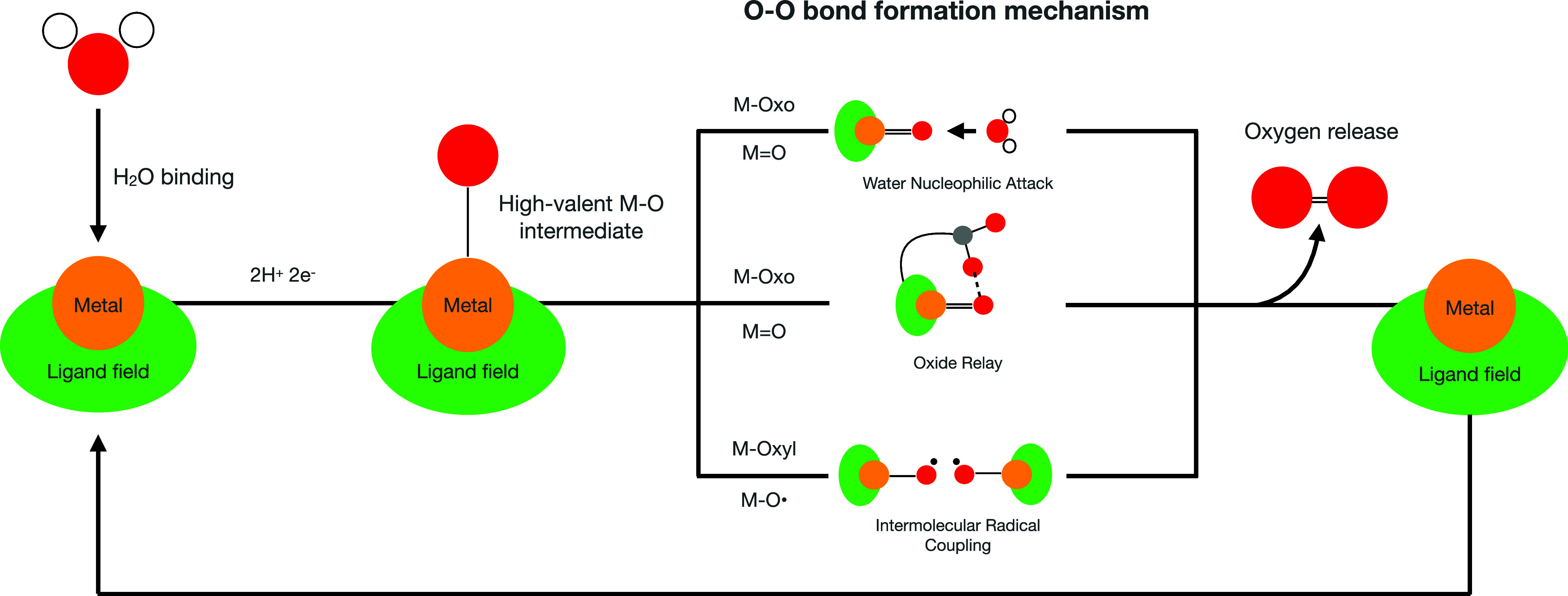
Schematic representation of the catalytic cycle in the water oxidation reaction. Also represented are the three possible pathways for O–O bond formation.

Molecular water oxidation catalysts (MWOCs) have been extensively studied in recent decades. The development of MWOCs is motivated by their high intrinsic activity, low overpotential, and high catalytic rate compared to heterogeneous catalysts.^1^ Among these catalysts, Ru-based complexes have set the benchmark for their superior performance and robustness, providing a profound understanding of the oxygen evolution catalytic cycle.^2−4^ A schematic general mechanism for water oxidation is presented in Figure 1.

**Figure 2:**
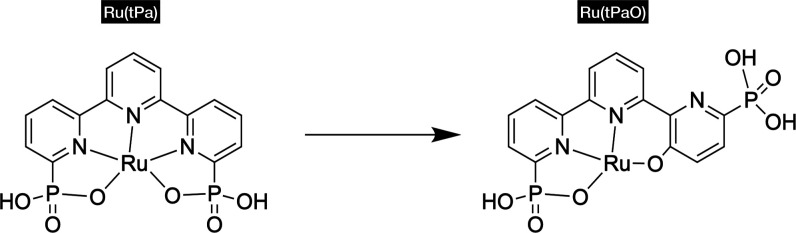
Schematic representation of the transformation of the equatorial ligand from tPa to tPaO.

The key step in the water oxidation reaction is the O–O bond formation, which is highly dependent on the electronic structure of the organometallic complex.^5^ The electronic structure of the complex is mainly determined by the combination of the metal’s d orbitals and the ligands’ s and p orbitals. When the metal center remains the same, the structure is solely determined by the choice of ligands. Specifically, the O–O bond formation mechanism depends on the nature of the high-valent M–O intermediate.^5^ If the oxygen has an oxo character, characterized by lower spin density and high electrophilicity,^6^ the pathway can involve either water nucleophilic attack (WNA)^7,8^ or the oxide relay (OR) mechanism.^9,10^ On the other hand, if the oxygen has an oxyl radical character, characterized by high spin density and low partial charge, the pathway can involve intermolecular radical coupling (I2M),^11^ provided the supramolecular properties allow for correct alignment.^12^ The nature of the high-valent intermediate not only influences the O–O bond formation pathway but also affects the catalyst’s reactivity and stability, which are essential factors in defining a good catalyst. The first Ru-based MWOC was developed by Meyer et al. in 1982^13^ with a turnover frequency (TOF) of 0.004 s^–1^ and a turnover number (TON) of 13. Subsequent developments did not significantly improve reactivity and stability until the introduction of carboxylate ligands by Sun and Åkermark in 2009, which achieved a TOF of 0.28 s^–1^ and a TON of 4700.^5,14^ The introduction of carboxylate groups was inspired by their stabilization effect on high-valent Mn states in binuclear Mn complexes.^15^ Building upon this milestone, Sun’s group developed Ru(bda)(py)_2_ (bda = 2,2′-bipyridine-6,6′-dicarboxylate, py = pyridine), which reached a TOF of 41 s^–1^ and a TON exceeding 2000 at pH 1 by undergoing the I2M mechanism for O–O bond formation. Llobet’s group developed the complex Ru(tda)(py)_2_, which can achieve a maximum TOF of 8000 s^–1^ (at pH 7) and was proposed to follow the WNA mechanism.^2^ Our group proposed that Ru(tda)(py)_2_ (tda = [2,2′:6′,2″-terpyridine]-6,6″-dicarboxylate) undergoes the OR mechanism,^9,10^ where the carboxylate acts as a nucleophile rather than a base. The OR mechanism is characterized by the donation of oxygen from the carboxylate ligand, followed by a hydroxide nucleophilic attack that breaks the percarboxylate intermediate and precedes the release of oxygen. In an experimental report, Mandal and collaborators^16^ synthesized and characterized a new ruthenium complex, [Ru(trpy)(H_2_L^2^-κ-N_2_)(OH_2_)]^2+^ (2Aq), and compared its catalytic activity with a previously reported aqua-coordinated complex, [Ru(trpy)(HL^1^)(OH_2_)]^2+^ (1Aq), where trpy = 2,2’:6′,2″-terpyridine, HL^1^ = 2-(2-pyridyl)benzimidazole, and H_2_L^2^ = 2-(pyridin-2-yl)-1*H*-benzo[*d*]imidazole-4-carboxylic acid. By comparing the catalytic performances of 1Aq and 2Aq, they observed an effect due to the presence of the dangling carboxylate group on water oxidation reactivity. Complex 2Aq exhibited a significantly higher catalytic rate than complex 1Aq. These observations indicated that the dangling carboxylate group in 2Aq could enhance the reaction rate by participating in the O–O bond formation, where the oxygen of the closely placed pendant carboxylate group reacts with Ru^V^=O to form the ruthenium(III) percarboxylate intermediate, subsequently attacked by water in upcoming steps. Through a labeling experiment with ^18^O-labeled water, they observed the incorporation of the oxygen isotope into the carboxylate group of 2Aq under catalytic conditions. The role of carboxylate groups is well-defined as charge stabilizers and facilitators of proton transfer processes.^5^ Nevertheless, other functional groups such as phosphonates^17,18^ or sulfonates^19−21^ have been successfully employed in MWOCs. Llobet’s group investigated the substitution of carboxylate groups in Ru(tda)(py)_2_ with phosphonate groups and reported a TOF of 16,000 s^–1^ at pH 7. Additionally, a change in the coordination environment from Ru(tPa)(py)_2_ (tPa = 2,2:6,2″-terpyridine-6,6″-diphosphonate) to Ru(tPaO)(py)_2_ [tPaO = 3-(hydroxo-[2,2:6,2″-terpyridine]-6,6″-diyl)bis(phosphonate)] was observed^22^ and identified as necessary for catalyst activation (see Figure 2).

## Results and Discussion

Intramolecular radical couplings in MWOCs have been identified and reported in the literature. For example, in photosystem II, the presence of metal-oxo and metal-oxyl radicals is necessary for O–O bond formation in the S4 state of the oxygen evolution complex.^23−25^ Based on this occurrence in nature, other MWOCs have been synthesized, where intramolecular radical couplings promote O–O bond formation.^26,27^ In this study, we test the OR mechanism in the Ru(tPaO)(py)_2_ complex and identify a variation of it that involves an intramolecular radical coupling, which we have denominated as radical OR. To test the OR mechanism in the Ru(tPaO)(py)_2_ complex, we will follow a similar scheme as presented in Figure 1. First, we will calculate the potential from Ru^III^(tPaO)(py)_2_ (**IIIa**) to Ru^V^(O)(tPaO)(py)_2_ (**V**). Next, we will calculate the O–O bond formation step, hydroxide nucleophilic attack, and O–O release.

### Catalyst Activation: From Ru^III^ to Ru^V^=O

**Figure 3:**
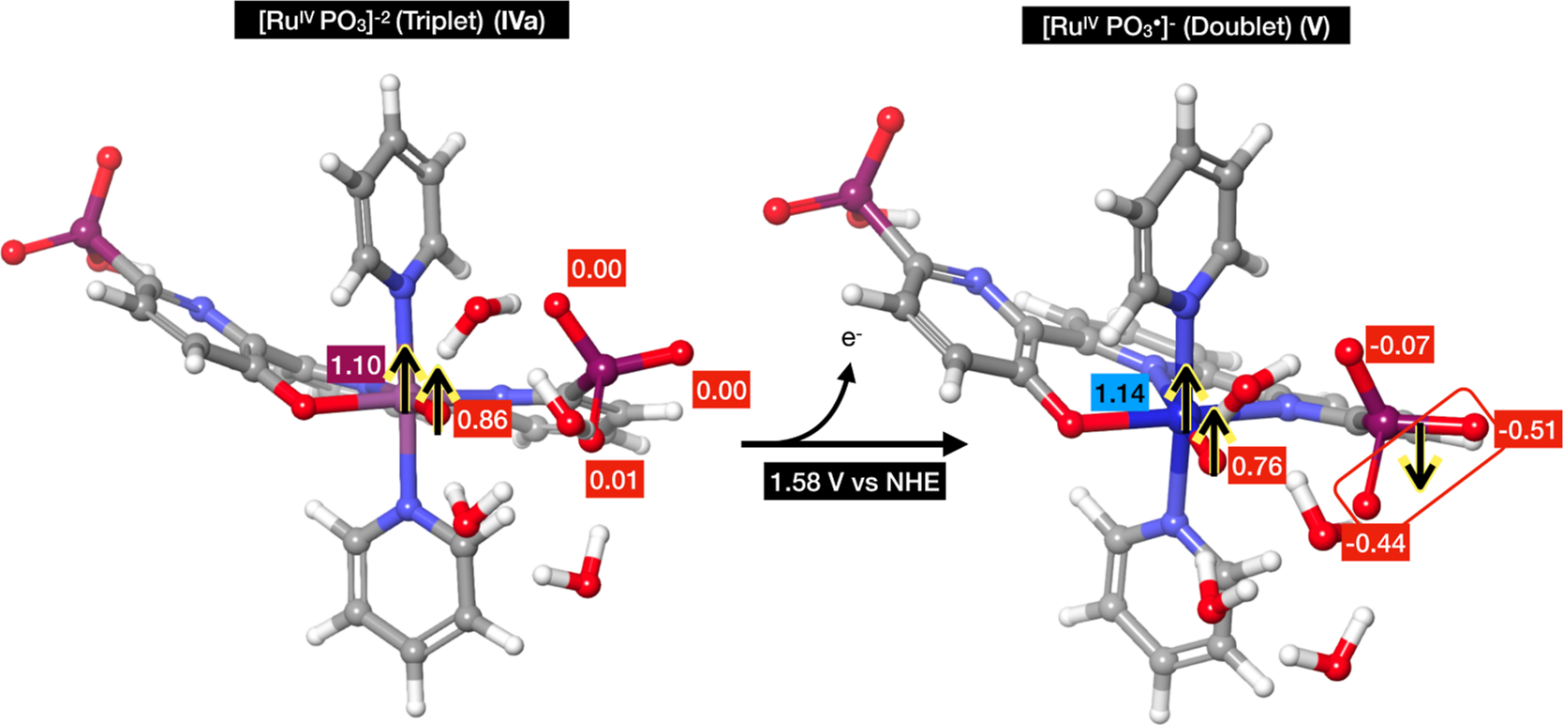
Spin density distribution in the **V** intermediate for selected atoms with the highest values. The blue and purple labels represent the Ru center, and the red labels represent the oxygen atoms. The arrows represent the spin.

This elementary step involves the coordination of water onto Ru^III^(tPaO)(py)_2_ (**IIIa**), followed by a two-electron oxidation and the release of two protons from water. Experimental characterization using cyclic voltammetry gives a potential of 1.4 V versus normal hydrogen electrode^22^ (NHE). We have computed the potential using density functional theory (DFT) at the B3LYP-D3^28,29^ level of theory (see computational methods in the Supporting Information for more details). The computed potential from the initial structure [Ru^III^(tPaO)(py)_2_]^1–^ (**IIIa**) to the reactive Ru^V^ = O (**V**) intermediate is 1.54 V versus NHE, which closely matches the experimental data. One remarkable feature of the reactive intermediate is the distribution of spin density. The Ru^V^ complex (**V**) is an open-shell doublet (the quartet energy was also computed and is almost identical, albeit slightly higher by 0.13 kcal/mol), with the spin distributed among the Ru center, the oxo ligand, and the oxygen atoms in the phosphonate, as shown in Figure 3.

The Ru center and the oxo together exhibit an alpha spin density of approximately 2, while the phosphonate oxygen atoms exhibit a slightly lower beta spin density, approaching −1. As a result, the total spin corresponds to a single alpha unpaired electron (doublet). The high spin values in the oxygen atoms bonded to the Ru center confer an alpha radical character (oxyl), and the beta densities in the phosphonate oxygen atoms also indicate significant radical character, suggesting that the high-valent intermediate **V** is a Ru^IV^–PO_3_^•^ species rather than a Ru^V^=O species. The beta spin appears to be shared between the two oxygen atoms in the phosphonate, indicating conjugation between these oxygen atoms. This radical character could significantly decrease the activation energy required for O–O bond formation;^30^ moreover, experimental evidence of intramolecular radical O–O bond formation between an oxyl and a N–O^•^ radical has been reported by Pushkar and collaborators.^31^ Since, at pH 7, the reported species contain a fully deprotonated noncoordinating phosphonate, the oxidation is not coupled to a proton transfer. We performed an optimization of the Ru^IV^ (**IVa**) intermediate and found that the triplet multiplicity is the lowest in energy. Analysis of the Mulliken spin densities reveals that the two unpaired alpha electrons are located in the oxyl and the Ru metal center (Figure 3, right side). Thus, the **IVa**/**V** oxidation removes an alpha electron from the phosphonate, leaving two oxygen atoms sharing the beta unpaired electron and reducing the spin multiplicity to a doublet. The potential for the **IVa**/**V** oxidation has been computed with a value of 1.58 V versus NHE, which is almost identical to the overall potential value from **IIIa** to **V**, indicating that this step determines the overpotential. Since 1.58 V appears to be a low potential for a phosphonate, we have verified whether the obtained potential can be explained by the coordination environment. To that end, we calculated the potentials (vs NHE) for a set of smaller models: methyl phosphonate (Me-phos), pyridyl phosphonate (py-phos), and terpyridyl phosphonate (tpy-phos). The reactions and their corresponding potentials are as follows
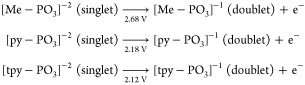


**Figure 4:**
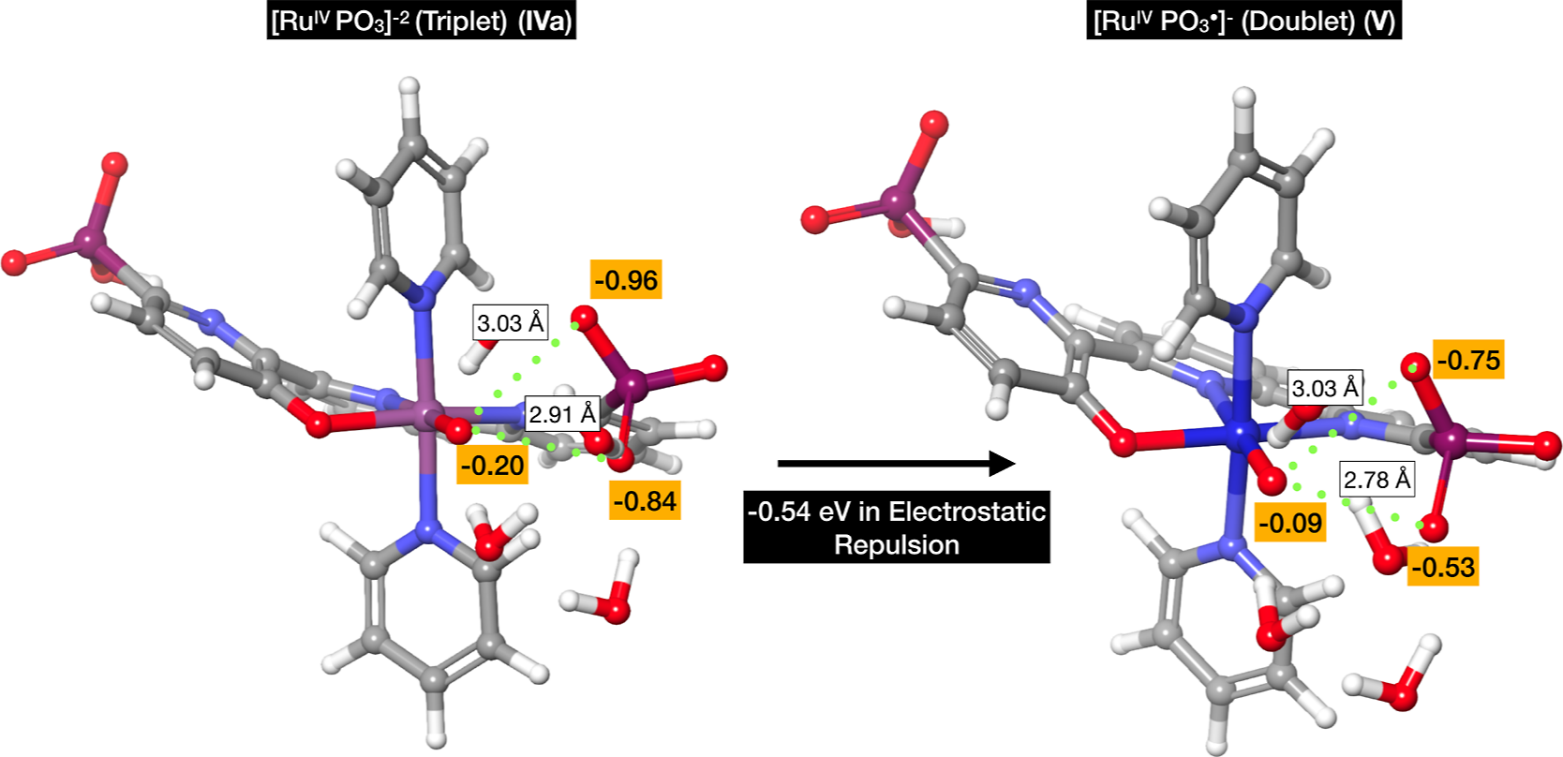
ESP charges for the oxyl and the oxygen atoms in the phosphonate and the distances in the **IVa** and **V** complexes.

We observed that the presence of conjugated pyridine reduces the potential, but it is still higher compared to the computed value for the complex. From the analysis of the partial charges and distances between the oxyl and the closest oxygen atoms in the phosphonate, we observe a reduction in the negative partial charges, which can alleviate some strain due to Coulomb repulsion. Specifically, by applying Coulomb’s law between the oxyl and the oxygen atoms in the phosphonate, we obtained a reduction in repulsion of 0.54 eV from Ru^IV^ (**IVa**) to Ru^IV^–PO_3_^•^ (**V**) (Figure 4). The strain induced by the higher nuclear repulsion in the Ru^IV^ complex (**IVa**) favors the oxidation of the phosphonate, reducing the potential to 1.58 V, which is accessible under experimental electrocatalytic conditions.

**Figure 5:**
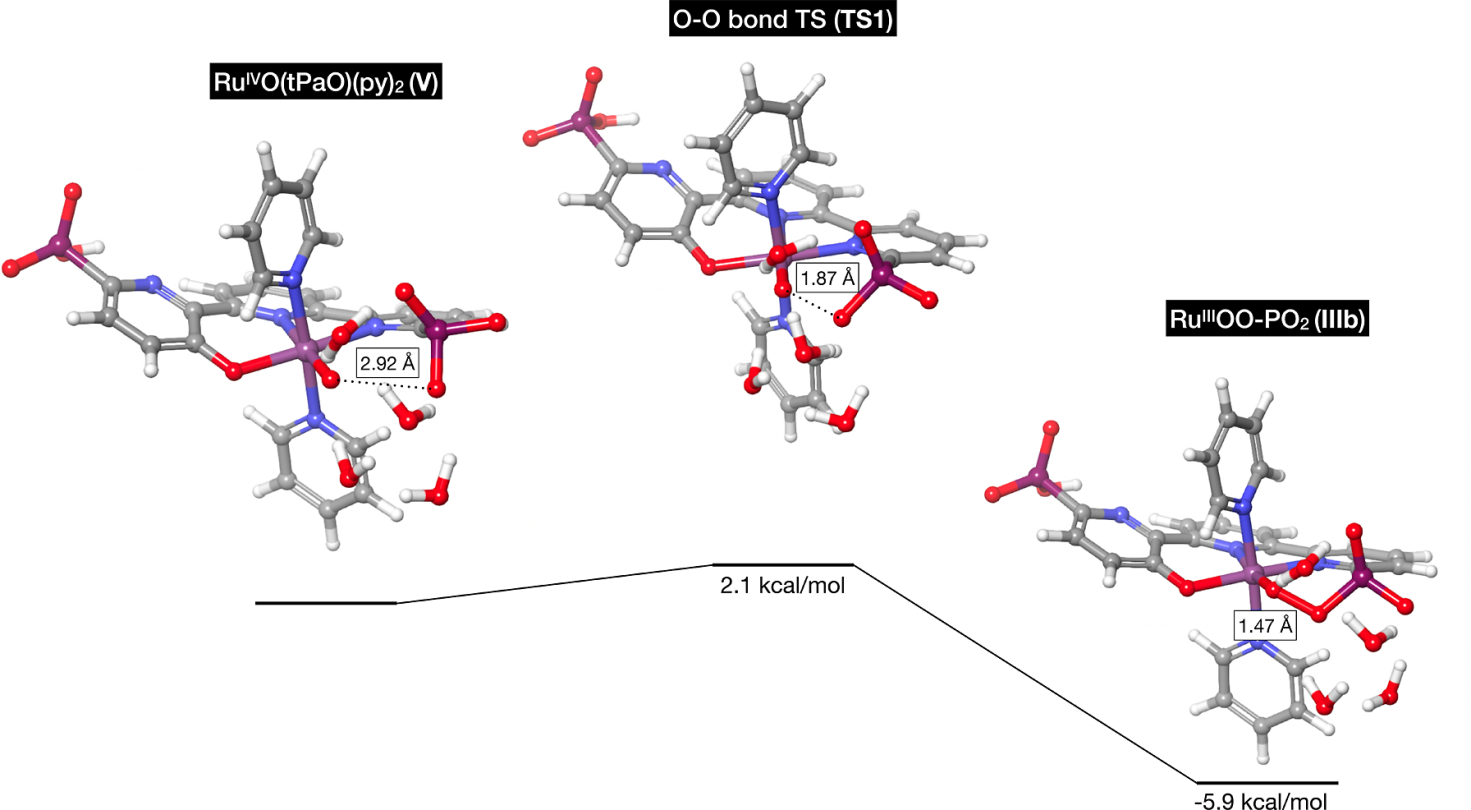
O–O bond formation energy profile computed by DFT, as well as the O–O distances during the reaction.

O–O bond formation is a critical step in all mechanisms of catalytic water oxidation. In the WNA mechanism, the barrier to O–O bond formation is attributed to enthalpic effects resulting from electronic structure reorganization. In the I2M mechanism, the barrier arises from the enthalpy penalty associated with the dimerization of two radical catalysts. In the OR mechanism involving Ru(tda)(py)_2_, O–O bond formation becomes the rate-limiting step for pH values above 8–9.^9^ It has an activation free energy of 10.8 kcal/mol, primarily driven by enthalpic contributions. Through spin density analysis, we observed a significant difference between Ru^IV^(O)(tPaO)(py)_2_ (**V**) and Ru^V^(O)(tda)(py)_2_. The oxygen ligand in the carboxylate complex exhibits low spin density, resembling an oxo character. Conversely, in the phosphonate complex, the oxygen demonstrates a radical oxo character with a spin density of 0.86. Additionally, the adjacent phosphonate oxygen displays significant spin density, suggesting the potential for intramolecular radical coupling. The activation free energy for O–O bond formation is remarkably low, at only 2.1 kcal/mol, indicating a nearly barrierless process compared to the Ru^V^(O)(tda)(py)_2_ species. The computed activation free energy is notably lower than that of the WNA pathway proposed by Llobet and collaborators, who reported a computed barrier of 22.1 kcal/mol. The barrier of the OR pathway is therefore better aligned with the experimentally measured TOF.^22^ Furthermore, the O–O bond formation reaction is exergonic with a reaction free energy of −5.9 kcal/mol (Figure 5). Due to the radical nature of the involved atoms and the absence of an entropic penalty resulting from intramolecular radical coupling, the O–O bond formation step demonstrates minimal enthalpic and entropic barriers, making it nearly barrierless in terms of free energy.

### Hydroxide Nucleophilic Attack and Oxygen Release

**Figure 6:**
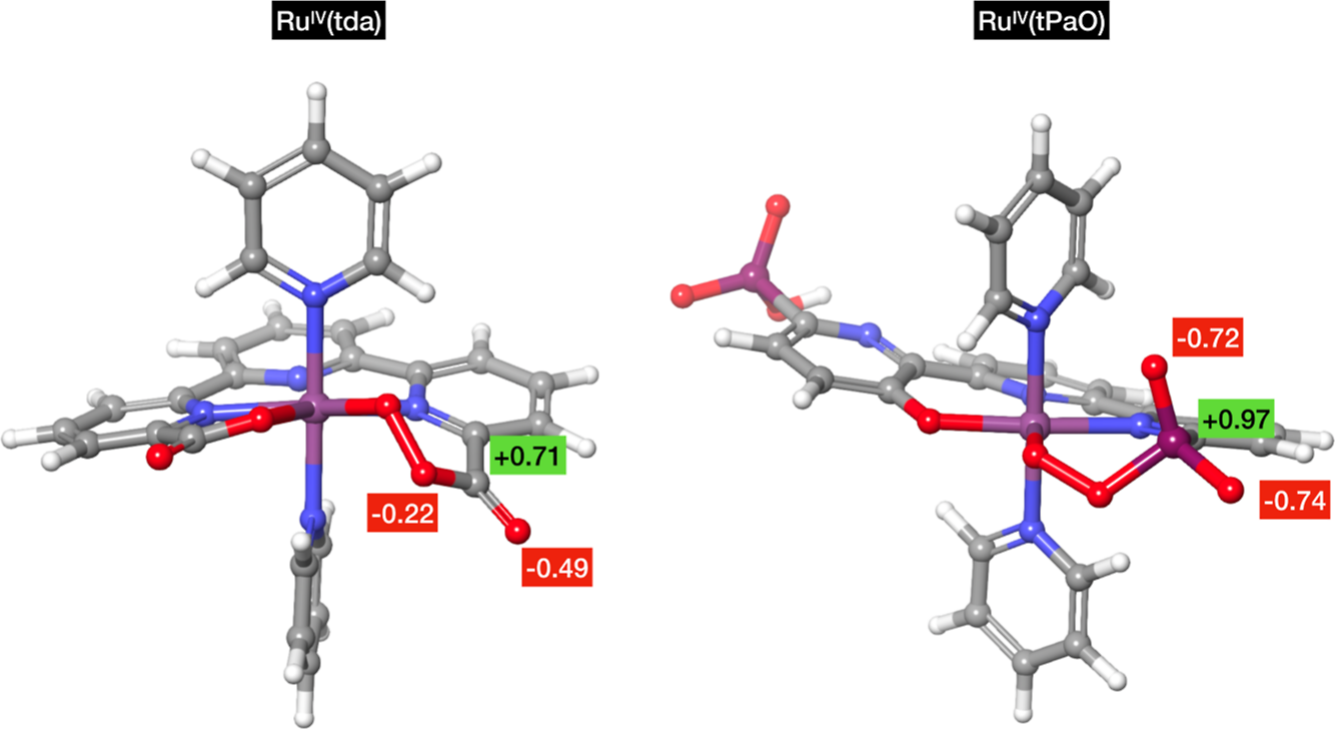
Comparison of the partial charges involved in the OH^–^ nucleophilic attack between Ru(tda) (left side) and Ru(tPaO) (**IVb**) (right side).

The hydroxide nucleophilic attack plays a crucial role in liberating O_2_ from the dangling ligand and regenerating the ligand. To facilitate this process, we proposed an oxidation step from Ru^III^ to Ru^IV^ in Ru(tda)(py)_2_ to enhance the electrophilicity of the carboxylate and promote the hydroxide attack.^9^ Similarly, we calculated the potential for the Ru^III^ intermediate (**IIIb**) to Ru^IV^ (**IVb**), obtaining a value of 1.46 V versus NHE. Although slightly higher than that of the carboxylate complex (1.29 V vs NHE), this potential remains reasonable under experimental electrocatalytic conditions. The Ru^III/IV^ oxidation results in a change in the multiplicity of the complex. The ground state of Ru^IV^ (**IVb**) is a triplet, whereas in Ru(tda), as a 7-coordinated complex, it is a singlet. Another significant observation in Ru(tda) is the positive partial charge (+0.7) on the carbon atom of the carboxylate in the Ru^IV^ percarboxylate intermediate. This positive partial charge facilitates the nucleophilic attack by promoting an attractive Coulomb interaction between the negatively charged hydroxide ion and the carbon atom. In the case of the phosphonate ligand, it exhibits greater steric hindrance compared to the carboxylate. However, the phosphorus atom in the phosphonate carries a partial charge of +0.97. Nevertheless, the Coulomb repulsion between the oxygen atoms in the phosphonate and the incoming hydroxide is stronger than that between the oxygen atoms in the carboxylate and the hydroxide (see Figure 6). Consequently, we anticipate a higher activation energy for the subsequent step compared to Ru(tda).

**Figure 7:**
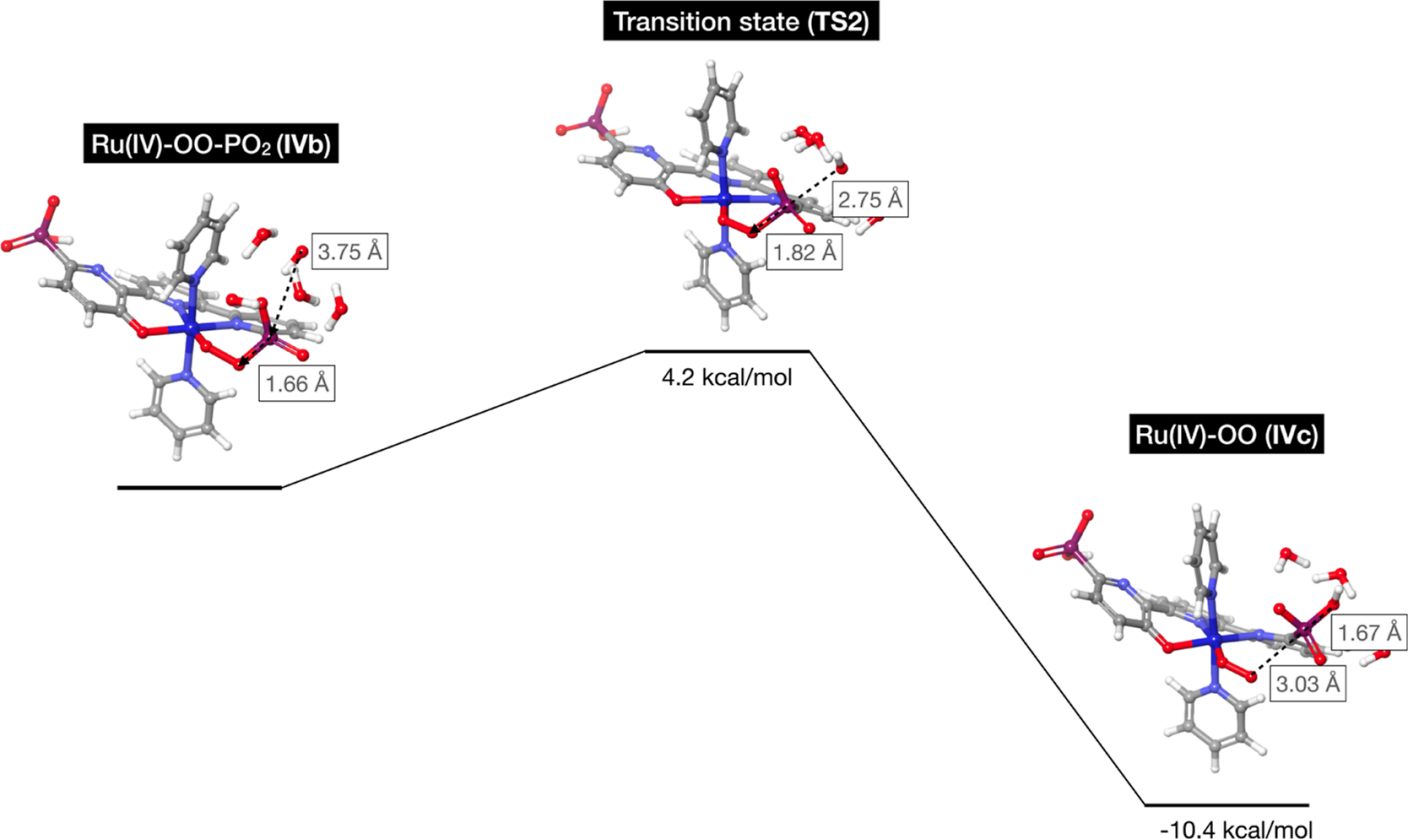
Hydroxide nucleophilic attack free energy profile computed by DFT. Also included are the HO–P distances and the P–OO distances during the reaction.

The OH^–^ nucleophilic attack serves as the crucial second step in the OR mechanism and is responsible for the pH-dependent reactivity of Ru(tda).^9,10^ As the reaction is likely to occur at the solvent–electrode interface, we anticipate a localized increase in OH^–^ concentration. Previous reports have indicated a pH increase of approximately 1.2 at the anode-solvent interface.^32^ Starting from the Ru^IV^ intermediate (**IVb**), we introduced an explicit OH^–^ and calculated the nucleophilic attack on the phosphorus atom, analogous to a phosphate hydrolysis reaction. The calculated transition state relative to the Ru^IV^ intermediate (**IVb**) has a Gibbs free energy of 4.2 kcal/mol. During the coordination of hydroxide to the phosphonate, the P–OO bond is simultaneously cleaved, and the transition state does not exhibit a trigonal bipyramidal pentacoordinated phosphonate. The reaction is also exergonic, with a reaction free energy of −10.4 kcal/mol (Figure 7). In the phosphonate complex, the oxygen release step is more straightforward compared to the carboxylate complex. This is due to Ru^IV^(OO)(tPaO)(py)_2_ (**IVc**) already being in a triplet state, eliminating the need for a singlet–triplet intersystem crossing as observed in Ru^IV^(OO)(tda)(py)_2_. The release of molecular oxygen (O_2_) is barrierless and exothermic, with a reaction free energy of −41.5 kcal/mol relative to Ru^IV^(OO)(tPaO)(py)_2_ (**IVc**).

## Conclusions

Our investigation focused on the viability of the OR mechanism in the Ru(tPaO)(py)_2_ molecular catalyst. Our findings indicate that this complex can indeed undergo the OR mechanism. However, in this specific complex, the two oxygen atoms involved in O–O bond formation both exhibit radical characters. Since two radicals typically combine with negligible activation energies the enthalpic contribution to the barrier is largely absent. Since the two radicals are part of the same molecule there are also negligible entropic contributions. Consequently, the reaction barrier for O–O bond formation is significantly lowered to only 2.1 kcal/mol. Such low activation energy indicates in an intramolecular reaction indicates that the O–O bond formation is never rate limiting, which is very rare in catalytic water oxidation. We have also identified the OH^–^ nucleophilic attack in the Ru(tPaO) complex as the more likely rate-limiting step under low pH, and its reactivity is strongly dependent on pH for the phosphonate-based complex. Electron transfer and ligand substitution steps have not been investigated, but could also limit the rate under some conditions. The unique characteristics of Ru(tPaO)(py)_2_ distinguish the radical OR mechanism from the previously reported OR mechanism. Notably, to the best of our knowledge, the radical OR mechanism represents the most efficient mechanism reported for O–O bond formation and opens up avenues for exploring highly efficient MWOCs.

## Supporting Information Available

All computational details and methods employed in this article, along with the energy components of intermediates within the catalytic cycle, including small models for phosphonate oxidation, and a comparison of the performance of several functionals in terms of redox potentials. Cartesian coordinates of all intermediates (PDF)
