# Supplementary material for: Removing the Barrier in O–O Bond Formation Via the Combination of Intramolecular Radical Coupling and the Oxide Relay Mechanism
Source: J Phys Chem A. 2024 May 6;128(19):3794–800. doi: 10.1021/acs.jpca.4c00404 (PMC11103688; doi:10.1021/acs.jpca.4c00404)
Supplement: Supplementary file 1 — jp4c00404_si_001.pdf [file jp4c00404_si_001.pdf]

Supporting Information

Removing the Barrier in O-O Bond Formation via the Combination of Intramolecular Radical Coupling and the Oxide Relay Mechanism

Juan Angel de Gracia Triviño[*,†,§], Mårten S.G. Ahlquist[*,§]

[†]*PDC Center for High-Performance Computing, School of Electrical Engineering and Computer Science, KTH Royal Institute of Technology, 10691 Stockholm, Sweden*
[§]*Division of Theoretical Chemistry and Biology, Department of Chemistry, Royal Institute of Technology, SE-114 28, Stockholm, Sweden*

E-mail: jadgt@kth.se, ahlqui@kth.se

Contents

S1 Computational Details **S1**

S2 Detailed DFT energies **S1**
S2.1 Comparison of functionals for the Redox Potentials S2

S3 Geometries **S3**
S3.1 1. RuIII - Doublet, Charge = -1 . . . . . S3
S3.2 1-2. RuIV - Triplet, Charge = -2 . . . . . S4
S3.3 2. RuIV - Open Shell Doublet, Charge = -2 . . . . . S5
S3.4 3. OO Bond TS - Doublet, Charge = -1 . . . . . S6
S3.5 4. RuIII-OO - Doublet, Charge = -1 . . . . . S7
S3.6 5. RuIV-OO - Triplet, Charge = 0 . . . . . S9
S3.7 6. RuIV-OO + OH- - Triplet, Charge = -1 . . . . . S10
S3.8 7. OH-attack-TS - Triplet, Charge = -1 . . . . . S11
S3.9 8. RuIV-OO - Triplet , Charge = -1 . . . . . S12
S3.109. OO-Release - Triplet, Charge = -1 . . . . . S14

S4 References **S16**

S1 Computational Details

All DFT calculations to estimate Gibbs free energies were performed using the Jaguar 8.3 program package developed by Schrödinger LLC[1]. Molecular geometries were optimized using the B3LYP functional, which combines Becke's three-parameter hybrid functional with the LYP correlation functional[2], and Grimme's D3 correction was applied[3,4]. The LACVP** basis set was used for the optimization[5]. Single-point energy corrections were performed using the B3LYP-D3 functional with the LACV3P**++ basis set, which includes two f-functions on the metal. Frequency calculations were conducted on the optimized geometries to confirm their nature as minima or transition states on the potential energy surface. To account for solvation effects, single-point calculations with the Poisson-Boltzmann reactive field method (PBF) implemented in Jaguar 8.3 were carried out in water. The Gibbs free energy was calculated using the following equation: G = E(B3LYP-D3/LACV3P**++ 2f on Ru) + Gsol + ZPE + H298 - TS298 + 1.9 kcal/mol (where 1.9 kcal/mol represents a concentration correction to the free energy of solvation, which is by default calculated at a concentration of 1 M (g) to 1 M (aq) in Jaguar).

S2 Detailed DFT energies

All the energies for all the steps including their components are specified in the following tables:

Step	E(LACV3P++**)	Gs (kcal/mol)	ZPE (kcal/mol)	H (kcal/mol)	S (cal/mol)	Total (kcal/mol)	Delta Gibbs
1 - RuIII + H2O (D)[-1]	-2847.882342	-116.4596	329.78	27.582	253.579	-1786904.565	Redox
1 to 2 - RuIV=O (T)[-1]	-2923.0874	-208.7833	330.38	28.493	263.993	-1834190.332	
2 - RuV=O (OSD)[-1]	-2923.009584	-122.4862	329.522	29.101	263.378	-1834055.271	0.0
3 - OO-bond-TS (D)[-1]	-2923.012433	-119.1076	330.094	28.511	261.763	-1834053.217	2.1
4 - RuIII-OO-PO2 (D)[-1]	-2923.040078	-111.7626	331.681	28.356	259.521	-1834061.119	-5.8
5 - RuIV-OO-PO2 (T)[0]	-2922.872588	-83.9033	330.401	27.947	256.424	-1833928.924	Redox
6 - RuIV-OO-PO2 + OH- (T)[-1]	-2998.812878	-114.7657	338.289	29.805	273.319	-1881608.291	0.0
7 - OH-attack-TS (T)[-1]	-2998.796844	-122.2513	338.341	29.382	266.613	-1881604.087	4.2
8 - RuIV-OO (T)[-1]	-2998.836012	-112.6971	339.358	29.551	269.16	-1881618.685	-10.4
9 - OO-release (T)[-1]	-2998.89643	-104.0964	339.809	30.067	278.402	-1881649.784	-41.5

Table S1: Energy components for the steps in the catalytic cycle. The letter between parenthesis indicates the spin multiplicity and the number in between square brackets indicate the complex charge. The Delta Gibbs column indicates the relative free energy.

**Step**	**Delta Gibbs (kcal/mol)**
1. RuIII + H2O > 2. RuV + 2H	268.1356478
2 electron process	134.0678239
Redox vs NHE	**1.54 V**
1 to 2 RuIV=O > 2 RuV=O	135.060852
Redox vs NHE	**1.58 V**
5. RuIII-OOP > 6. RuIV-OOP	132.195
Redox vs NHE	**1.46 V**

Table S2: Oxidation potentials vs NHE (4.28 V) computed from the energies in Table S1

Additionally, the solvated proton free energy has been calculated and corrected with the pH value (in this case pH 7.0) according to Table S3:

**Proton Free Energy**		
Proton G (1 atm) in gas phase	-6.280	kcal/mol
Solvation G (1 atm to 1M)	-264.000	kcal/mol
pH correction (RT ln(C1/C2)	-9.558	kcal/mol
pH	7	
C2	0.0000001	
Proton Free Energy	-279.838	kcal/mol

Table S3: Free energy components of the solvated proton free energy

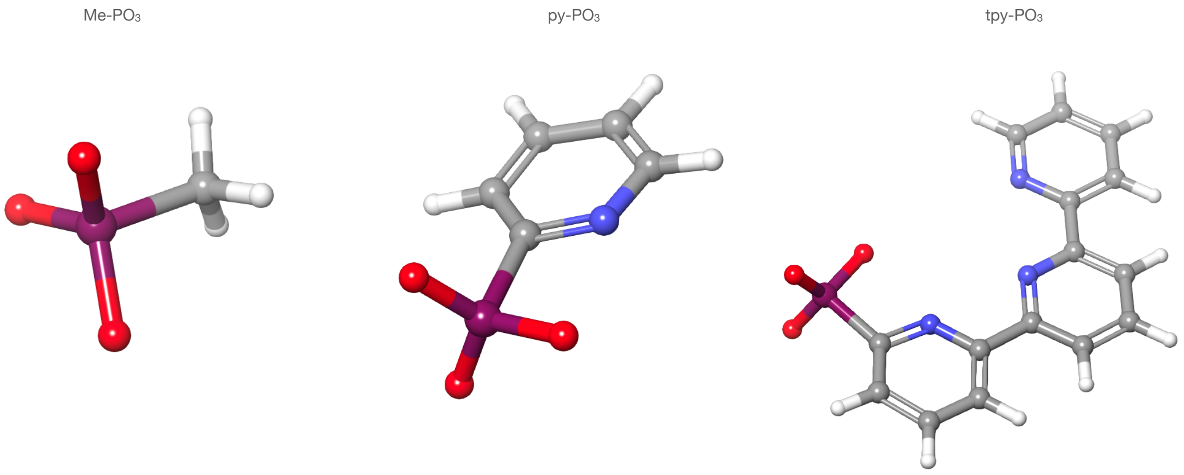

Figure S1: Models for the phosphonate oxidation considered in this study.

The phosphonate oxidation for the models (figure S1) has been calculated using the 6-311G++**[6] basis set and, due to the simplicity of these systems, the free energy has been calculated as G=G(6-311G**++) + G_{solv}
The free energies and redox potentials from are given in Table S4:

Table S4: Redox potentials and Gibbs free energies for different models of phosphonates

Model	Basis Set	G (kcal/mol)	Gs (kcal/mol)	Total	ΔG (kcal/mol) and V vs NHE
Methyl phosphonate (S)[-2]	6-311G++**	-607.028675	-263.42	-381179.3768	160.3088012
Methyl phosphonate (D)[-1]	6-311G++**	-607.0599705	-83.473	-381019.068	2.68
Pyril phosphonate (S)[-2]	6-311G++**	-814.8729227	-256.4794	-511596.5723	148.8043058
Pyril phosphonate (D)[-1]	6-311G++**	-814.9338447	-69.446	-511447.768	2.18
Terpyril phosphonate (S)[-2]	6-311G++**	-1309.165341	-261.1151	-821774.1493	147.4018508
Terpyril phosphonate (D)[-1]	6-311G++**	-1309.213634	-83.409	-821626.7475	2.12

S2.1 Comparison of functionals for the Redox Potentials

	B3LYP-D3	M11[7]	PBE0[8]	ωb97m-v[9]	Expt.10
1 - 2	1.49 V	1.69 V	1.57 V	1.36 V	
2 - 3	1.58 V	1.85 V	1.24 V	1.51 V	
1 - 3	1.54 V	1.77 V	1.40 V	1.44 V	1.4 V

Table S5: Redox potentials between the state 1 (Ru^{III} + H_2O) state 2 (Ru^{IV} + $2H^+$) and state 3 (Ru^{IV} open shell doublet) with different functionals and the experimental data.

Based on the benchmark in Table S5, we have utilized the hybrid functional B3LYP with empirical dispersion (D3) to achieve reasonable agreement with the range-separated hybrid, meta-GGA functional ωB97M and the experimental values. The ωB97M-V functional has been recently benchmarked against transition metal databases with remarkable success [11,12], and it will be used as the computational reference.

S3 Geometries

S3.1 1. RuIII - Doublet, Charge = -1

O1	-4.7848000000000	1.0312000000000	0.0284000000000
Ru2	-0.5988000000000	-0.1177000000000	-0.0793000000000
C3	2.6856000000000	-0.4887000000000	-0.3073000000000
C4	2.0099000000000	0.2599000000000	-1.3174000000000
C5	2.8038000000000	1.0665000000000	-2.1625000000000
C6	4.1699000000000	1.1357000000000	-1.9748000000000
C7	4.7579000000000	0.3803000000000	-0.9412000000000
H8	2.3010000000000	1.6249000000000	-2.9465000000000
H9	4.7981000000000	1.7509000000000	-2.6103000000000
C10	2.0339000000000	-1.4459000000000	0.6197000000000
C11	2.8100000000000	-2.4270000000000	1.2670000000000
C12	2.2213000000000	-3.3043000000000	2.1625000000000
H13	3.8676000000000	-2.4679000000000	1.0462000000000
C14	0.1170000000000	-2.2145000000000	1.7958000000000
C15	0.8583000000000	-3.1894000000000	2.4493000000000
H16	2.8172000000000	-4.0663000000000	2.6535000000000
H17	0.3910000000000	-3.8394000000000	3.1783000000000
C18	-1.2971000000000	-1.9144000000000	2.0939000000000
C19	-2.1079000000000	-2.5762000000000	3.0173000000000
C20	-3.0356000000000	-0.3694000000000	1.6120000000000
C21	-3.4111000000000	-2.1187000000000	3.2200000000000
H22	-1.7358000000000	-3.4308000000000	3.5688000000000
C23	-3.8800000000000	-1.0029000000000	2.5227000000000
H24	-4.0545000000000	-2.6300000000000	3.9284000000000
H25	-4.8864000000000	-0.6282000000000	2.6740000000000
N26	0.7035000000000	-1.3884000000000	0.8687000000000
N27	-1.7932000000000	-0.8502000000000	1.4149000000000
N28	4.0239000000000	-0.4079000000000	-0.1555000000000
C29	0.3638000000000	1.4433000000000	2.3644000000000
C30	0.8469000000000	2.5247000000000	3.0910000000000
C31	1.1531000000000	3.7086000000000	2.4187000000000
C32	0.9643000000000	3.7647000000000	1.0377000000000
C33	0.4780000000000	2.6425000000000	0.3787000000000
H34	0.1083000000000	0.5096000000000	2.8499000000000
H35	0.9750000000000	2.4323000000000	4.1636000000000
H36	1.5302000000000	4.5700000000000	2.9605000000000
H37	1.1856000000000	4.6617000000000	0.4706000000000
H38	0.3262000000000	2.6334000000000	-0.6933000000000
C39	-2.6920000000000	-1.7569000000000	-1.5959000000000
C40	-3.2155000000000	-2.7259000000000	-2.4436000000000
C41	-2.3563000000000	-3.6692000000000	-3.0045000000000
C42	-0.9947000000000	-3.5963000000000	-2.7026000000000
C43	-0.5427000000000	-2.5894000000000	-1.8586000000000
H44	-3.3346000000000	-1.0075000000000	-1.1619000000000
H45	-4.2781000000000	-2.6968000000000	-2.6531000000000
H46	-2.7342000000000	-4.4410000000000	-3.6677000000000
H47	-0.2836000000000	-4.3027000000000	-3.1166000000000
H48	0.5079000000000	-2.4902000000000	-1.6163000000000
N49	-1.3782000000000	-1.6828000000000	-1.3073000000000
N50	0.1845000000000	1.4985000000000	1.0294000000000
P51	-3.3958000000000	1.1720000000000	0.6475000000000
P52	6.5588000000000	0.4809000000000	-0.5764000000000
O53	-2.2546000000000	1.0750000000000	-0.4903000000000
O54	6.7375000000000	1.1899000000000	0.7481000000000
O55	6.9131000000000	-1.1374000000000	-0.3977000000000
O56	-3.1434000000000	2.3581000000000	1.5635000000000
O57	7.2745000000000	0.9459000000000	-1.8217000000000

O58	0.7048000000000	0.2110000000000	-1.5511000000000
H59	6.3723000000000	-1.4882000000000	0.3307000000000
O60	-4.7760000000000	3.1827000000000	-1.8236000000000
H61	-4.0165000000000	3.6482000000000	-1.4008000000000
O62	-2.3908000000000	3.9972000000000	-0.5553000000000
H63	-2.6330000000000	3.6454000000000	0.3364000000000
H64	-2.0019000000000	3.1931000000000	-0.9442000000000
O65	-3.2561000000000	1.2684000000000	-3.1091000000000
H66	-2.8078000000000	1.0711000000000	-2.2597000000000
H67	-3.8158000000000	2.0419000000000	-2.8474000000000
O68	-5.4367000000000	-0.3602000000000	-2.3210000000000
H69	-4.7037000000000	0.0991000000000	-2.7872000000000
H70	-5.3487000000000	0.0238000000000	-1.4235000000000
H71	-4.9708000000000	2.4669000000000	-1.1762000000000

S3.2 1-2. RuIV - Triplet, Charge = -2

O1	4.3330000000000	-1.0408000000000	-0.2507000000000
O2	1.6035000000000	-0.9904000000000	-1.2514000000000
Ru3	0.5365000000000	0.0536000000000	-0.2637000000000
C4	-2.8270000000000	0.5763000000000	-0.2937000000000
C5	-2.2134000000000	-0.1062000000000	-1.3840000000000
C6	-3.0729000000000	-0.8635000000000	-2.2196000000000
C7	-4.4235000000000	-0.9467000000000	-1.9484000000000
C8	-4.9412000000000	-0.2669000000000	-0.8280000000000
H9	-2.6264000000000	-1.3728000000000	-3.0689000000000
H10	-5.0870000000000	-1.5227000000000	-2.5864000000000
C11	-2.1076000000000	1.4986000000000	0.6108000000000
C12	-2.8171000000000	2.5285000000000	1.2534000000000
C13	-2.1523000000000	3.3856000000000	2.1155000000000
H14	-3.8740000000000	2.6379000000000	1.0529000000000
C15	-0.1213000000000	2.1710000000000	1.7030000000000
C16	-0.7913000000000	3.2029000000000	2.3594000000000
H17	-2.6875000000000	4.1912000000000	2.6077000000000
H18	-0.2800000000000	3.8501000000000	3.0587000000000
C19	1.2988000000000	1.8343000000000	1.9608000000000
C20	2.0750000000000	2.6138000000000	2.8151000000000
C21	3.0275000000000	0.2405000000000	1.7340000000000
C22	3.3672000000000	2.2034000000000	3.1287000000000
H23	1.6807000000000	3.5279000000000	3.2386000000000
C24	3.8189000000000	0.9990000000000	2.6164000000000
H25	3.9893000000000	2.8055000000000	3.7839000000000
H26	4.7882000000000	0.5940000000000	2.8777000000000
N27	-0.7776000000000	1.3619000000000	0.8315000000000
N28	1.7982000000000	0.6936000000000	1.3794000000000
N29	-4.1521000000000	0.4744000000000	-0.0480000000000
C30	-0.3813000000000	-1.6344000000000	2.1004000000000
C31	-0.9573000000000	-2.6983000000000	2.7828000000000
C32	-1.4869000000000	-3.7629000000000	2.0516000000000
C33	-1.4189000000000	-3.7211000000000	0.6594000000000
C34	-0.8329000000000	-2.6195000000000	0.0467000000000
H35	0.0520000000000	-0.7937000000000	2.6262000000000
H36	-0.9826000000000	-2.6869000000000	3.8668000000000
H37	-1.9422000000000	-4.6084000000000	2.5576000000000
H38	-1.8130000000000	-4.5249000000000	0.0477000000000
H39	-0.7640000000000	-2.5334000000000	-1.0293000000000
C40	2.6608000000000	1.7804000000000	-1.5065000000000
C41	3.2601000000000	2.8193000000000	-2.2084000000000
C42	2.4644000000000	3.8616000000000	-2.6846000000000
C43	1.0874000000000	3.8183000000000	-2.4550000000000
C44	0.5531000000000	2.7385000000000	-1.7607000000000

H45	3.2378000000000	0.9459000000000	-1.1252000000000
H46	4.3294000000000	2.7663000000000	-2.3756000000000
H47	2.9045000000000	4.6895000000000	-3.2320000000000
H48	0.4283000000000	4.6014000000000	-2.8133000000000
H49	-0.5104000000000	2.6480000000000	-1.5766000000000
N50	1.3300000000000	1.7441000000000	-1.2892000000000
N51	-0.3301000000000	-1.5911000000000	0.7555000000000
P52	3.7988000000000	-1.3861000000000	1.1671000000000
P53	-6.7042000000000	-0.4069000000000	-0.3169000000000
O54	2.6518000000000	-2.4122000000000	1.2073000000000
O55	-6.8368000000000	-1.5654000000000	0.6507000000000
O56	-6.8403000000000	1.0364000000000	0.5029000000000
O57	4.9000000000000	-1.6197000000000	2.1998000000000
O58	-7.5902000000000	-0.3364000000000	-1.5403000000000
O59	-0.9347000000000	-0.0522000000000	-1.6959000000000
H60	-5.9550000000000	1.2194000000000	0.8757000000000
O61	4.4802000000000	-3.0586000000000	-2.0025000000000
H62	3.6237000000000	-3.4750000000000	-1.7373000000000
O63	1.9765000000000	-3.6914000000000	-1.0024000000000
H64	2.2474000000000	-3.2870000000000	-0.1192000000000
H65	1.6753000000000	-2.8681000000000	-1.4325000000000
O66	3.3942000000000	-1.0057000000000	-3.4407000000000
H67	2.7673000000000	-0.8256000000000	-2.7102000000000
H68	3.8419000000000	-1.8212000000000	-3.0899000000000
O69	5.4739000000000	0.4738000000000	-2.2092000000000
H70	4.7949000000000	0.0849000000000	-2.8044000000000
H71	5.2246000000000	0.0314000000000	-1.3658000000000
H72	4.5358000000000	-2.3335000000000	-1.3167000000000

S3.3 2. RuIV - Open Shell Doublet, Charge = -2

O1	4.3523000000000	-1.0759000000000	-0.2857000000000
O2	1.7123000000000	-0.9638000000000	-1.1339000000000
Ru3	0.5504000000000	0.0595000000000	-0.2271000000000
C4	-2.8125000000000	0.5325000000000	-0.2557000000000
C5	-2.1861000000000	-0.1575000000000	-1.3330000000000
C6	-3.0277000000000	-0.9254000000000	-2.1727000000000
C7	-4.3826000000000	-1.0029000000000	-1.9207000000000
C8	-4.9166000000000	-0.3069000000000	-0.8177000000000
H9	-2.5678000000000	-1.4440000000000	-3.0088000000000
H10	-5.0415000000000	-1.5840000000000	-2.5577000000000
C11	-2.1058000000000	1.4499000000000	0.6634000000000
C12	-2.8330000000000	2.4499000000000	1.3361000000000
C13	-2.1812000000000	3.3083000000000	2.2047000000000
H14	-3.8952000000000	2.5291000000000	1.1511000000000
C15	-0.1270000000000	2.1517000000000	1.7539000000000
C16	-0.8114000000000	3.1565000000000	2.4313000000000
H17	-2.7308000000000	4.0910000000000	2.7170000000000
H18	-0.3085000000000	3.8054000000000	3.1351000000000
C19	1.2994000000000	1.8366000000000	2.0019000000000
C20	2.0769000000000	2.6084000000000	2.8690000000000
C21	3.0569000000000	0.2966000000000	1.7252000000000
C22	3.3782000000000	2.2199000000000	3.1531000000000
H23	1.6717000000000	3.5047000000000	3.3187000000000
C24	3.8580000000000	1.0378000000000	2.6004000000000
H25	3.9976000000000	2.8168000000000	3.8141000000000
H26	4.8484000000000	0.6710000000000	2.8359000000000
N27	-0.7721000000000	1.3404000000000	0.8724000000000
N28	1.8100000000000	0.7216000000000	1.3970000000000
N29	-4.1413000000000	0.4388000000000	-0.0303000000000
C30	-0.3297000000000	-1.6324000000000	2.1557000000000

C31	-0.8789000000000	-2.7068000000000	2.8430000000000
C32	-1.3818000000000	-3.7876000000000	2.1169000000000
C33	-1.3171000000000	-3.7512000000000	0.7244000000000
C34	-0.7591000000000	-2.6396000000000	0.1051000000000
H35	0.0799000000000	-0.7788000000000	2.6796000000000
H36	-0.9044000000000	-2.6901000000000	3.9266000000000
H37	-1.8154000000000	-4.6416000000000	2.6275000000000
H38	-1.6924000000000	-4.5670000000000	0.1170000000000
H39	-0.6964000000000	-2.5583000000000	-0.9718000000000
C40	2.5800000000000	1.8697000000000	-1.5558000000000
C41	3.1051000000000	2.9200000000000	-2.2975000000000
C42	2.2426000000000	3.9088000000000	-2.7709000000000
C43	0.8776000000000	3.7988000000000	-2.4981000000000
C44	0.4205000000000	2.7096000000000	-1.7654000000000
H45	3.2147000000000	1.0777000000000	-1.1834000000000
H46	4.1697000000000	2.9192000000000	-2.4986000000000
H47	2.6225000000000	4.7450000000000	-3.3493000000000
H48	0.1674000000000	4.5374000000000	-2.8529000000000
H49	-0.6314000000000	2.5711000000000	-1.5507000000000
N50	1.2614000000000	1.7668000000000	-1.2949000000000
N51	-0.2777000000000	-1.5963000000000	0.8096000000000
P52	3.7510000000000	-1.3135000000000	1.1291000000000
P53	-6.6921000000000	-0.4368000000000	-0.3553000000000
O54	2.7195000000000	-2.3926000000000	1.3458000000000
O55	-6.8077000000000	-1.3508000000000	0.8457000000000
O56	-6.9795000000000	1.1386000000000	0.1060000000000
O57	5.0911000000000	-1.4934000000000	1.9124000000000
O58	-7.5064000000000	-0.6853000000000	-1.6030000000000
O59	-0.9003000000000	-0.0984000000000	-1.6383000000000
H60	-6.2836000000000	1.3931000000000	0.7378000000000
O61	4.4528000000000	-3.1364000000000	-2.1944000000000
H62	3.6199000000000	-3.5238000000000	-1.8279000000000
O63	2.0257000000000	-3.6998000000000	-0.9850000000000
H64	2.2889000000000	-3.4800000000000	-0.0615000000000
H65	1.7608000000000	-2.8045000000000	-1.2814000000000
O66	3.3129000000000	-0.9615000000000	-3.4594000000000
H67	2.6845000000000	-0.8147000000000	-2.7231000000000
H68	3.7471000000000	-1.8069000000000	-3.1795000000000
O69	5.3696000000000	0.6037000000000	-2.3827000000000
H70	4.6693000000000	0.1360000000000	-2.8956000000000
H71	5.2806000000000	0.1745000000000	-1.5140000000000
H72	4.6777000000000	-2.4842000000000	-1.4994000000000

S3.4 3. OO Bond TS - Doublet, Charge = -1

O1	-3.5682000000000	-0.9719000000000	0.5667000000000
O2	-1.7311000000000	-1.1257000000000	0.9018000000000
Ru3	-0.6103000000000	0.0892000000000	0.1385000000000
C4	2.7558000000000	0.4953000000000	0.2677000000000
C5	2.1029000000000	-0.2376000000000	1.3008000000000
C6	2.9251000000000	-1.0284000000000	2.1404000000000
C7	4.2850000000000	-1.0951000000000	1.9215000000000
C8	4.8460000000000	-0.3622000000000	0.8545000000000
H9	2.4438000000000	-1.5753000000000	2.9458000000000
H10	4.9290000000000	-1.6950000000000	2.5563000000000
C11	2.0767000000000	1.4544000000000	-0.6258000000000
C12	2.8315000000000	2.4611000000000	-1.2590000000000
C13	2.2091000000000	3.3649000000000	-2.1009000000000
H14	3.8933000000000	2.5074000000000	-1.0621000000000
C15	0.1229000000000	2.2425000000000	-1.7146000000000
C16	0.8379000000000	3.2528000000000	-2.3463000000000

H17	2.7819000000000	4.1521000000000	-2.5795000000000
H18	0.3537000000000	3.9371000000000	-3.0299000000000
C19	-1.3032000000000	1.9653000000000	-1.9942000000000
C20	-2.0617000000000	2.7336000000000	-2.8794000000000
C21	-3.0995000000000	0.4632000000000	-1.6930000000000
C22	-3.3711000000000	2.3598000000000	-3.1616000000000
H23	-1.6384000000000	3.6132000000000	-3.3460000000000
C24	-3.8852000000000	1.2068000000000	-2.5777000000000
H25	-3.9735000000000	2.9560000000000	-3.8390000000000
H26	-4.8923000000000	0.8649000000000	-2.7819000000000
N27	0.7405000000000	1.3860000000000	-0.8467000000000
N28	-1.8424000000000	0.8650000000000	-1.3911000000000
N29	4.0912000000000	0.4100000000000	0.0752000000000
C30	0.2259000000000	-1.4212000000000	-2.3882000000000
C31	0.7686000000000	-2.4367000000000	-3.1645000000000
C32	1.3092000000000	-3.5567000000000	-2.5312000000000
C33	1.2847000000000	-3.6194000000000	-1.1381000000000
C34	0.7277000000000	-2.5641000000000	-0.4271000000000
H35	-0.2089000000000	-0.5400000000000	-2.8410000000000
H36	0.7608000000000	-2.3441000000000	-4.2446000000000
H37	1.7399000000000	-4.3652000000000	-3.1130000000000
H38	1.6891000000000	-4.4695000000000	-0.6007000000000
H39	0.6938000000000	-2.5616000000000	0.6548000000000
C40	-2.5813000000000	1.7783000000000	1.7124000000000
C41	-3.0494000000000	2.7424000000000	2.5966000000000
C42	-2.1506000000000	3.6653000000000	3.1293000000000
C43	-0.8038000000000	3.5757000000000	2.7704000000000
C44	-0.4011000000000	2.5704000000000	1.9007000000000
H45	-3.2397000000000	1.0364000000000	1.2866000000000
H46	-4.1002000000000	2.7319000000000	2.8605000000000
H47	-2.4873000000000	4.4343000000000	3.8172000000000
H48	-0.0652000000000	4.2651000000000	3.1639000000000
H49	0.6368000000000	2.4502000000000	1.6202000000000
N50	-1.2797000000000	1.6916000000000	1.3729000000000
N51	0.2126000000000	-1.4807000000000	-1.0420000000000
P52	-3.7895000000000	-1.1264000000000	-1.0356000000000
P53	6.6325000000000	-0.4845000000000	0.4319000000000
O54	-2.9513000000000	-2.2407000000000	-1.6318000000000
O55	6.7720000000000	-1.3775000000000	-0.7821000000000
O56	6.9344000000000	1.0974000000000	0.0062000000000
O57	-5.2865000000000	-1.0762000000000	-1.2293000000000
O58	7.4150000000000	-0.7569000000000	1.6946000000000
O59	0.8097000000000	-0.2038000000000	1.5678000000000
H60	6.2683000000000	1.3612000000000	-0.6527000000000
O61	-4.2300000000000	-3.2983000000000	2.0946000000000
H62	-3.5193000000000	-3.6982000000000	1.5341000000000
O63	-2.1061000000000	-3.8644000000000	0.4085000000000
H64	-2.4547000000000	-3.4570000000000	-0.4249000000000
H65	-1.7285000000000	-3.0646000000000	0.8194000000000
O66	-2.8505000000000	-1.3536000000000	3.5211000000000
H67	-2.3300000000000	-1.1040000000000	2.7333000000000
H68	-3.2980000000000	-2.1767000000000	3.2047000000000
O69	-5.1244000000000	0.1343000000000	2.7559000000000
H70	-4.3323000000000	-0.2999000000000	3.1492000000000
H71	-5.0263000000000	-0.1022000000000	1.8195000000000
H72	-4.4202000000000	-2.4868000000000	1.5882000000000

S3.5 4. RuIII-OO - Doublet, Charge = -1

O1	-3.1018000000000	0.8894000000000	-0.8330000000000
O2	-1.6895000000000	1.2765000000000	-0.9425000000000

Ru3	−0.4510000000000	−0.0230000000000	−0.1425000000000
C4	2.8536000000000	−0.4583000000000	−0.2819000000000
C5	2.2092000000000	0.2322000000000	−1.3543000000000
C6	3.0450000000000	0.9487000000000	−2.2463000000000
C7	4.4080000000000	0.9944000000000	−2.0452000000000
C8	4.9606000000000	0.3087000000000	−0.9434000000000
H9	2.5687000000000	1.4590000000000	−3.0783000000000
H10	5.0603000000000	1.5398000000000	−2.7195000000000
C11	2.1721000000000	−1.3375000000000	0.6941000000000
C12	2.9253000000000	−2.2943000000000	1.4042000000000
C13	2.3130000000000	−3.1012000000000	2.3465000000000
H14	3.9820000000000	−2.3750000000000	1.1919000000000
C15	0.2325000000000	−1.9976000000000	1.8914000000000
C16	0.9498000000000	−2.9444000000000	2.6119000000000
H17	2.8887000000000	−3.8443000000000	2.8882000000000
H18	0.4720000000000	−3.5465000000000	3.3734000000000
C19	−1.1869000000000	−1.6788000000000	2.1507000000000
C20	−1.9576000000000	−2.3421000000000	3.1103000000000
C21	−2.9905000000000	−0.2587000000000	1.6341000000000
C22	−3.2754000000000	−1.9529000000000	3.3209000000000
H23	−1.5374000000000	−3.1586000000000	3.6829000000000
C24	−3.7957000000000	−0.8950000000000	2.5786000000000
H25	−3.8860000000000	−2.4687000000000	4.0543000000000
H26	−4.8193000000000	−0.5627000000000	2.7054000000000
N27	0.8401000000000	−1.2385000000000	0.9297000000000
N28	−1.7156000000000	−0.6568000000000	1.4184000000000
N29	4.1941000000000	−0.3953000000000	−0.1133000000000
C30	0.5958000000000	1.6683000000000	2.1870000000000
C31	1.1406000000000	2.7701000000000	2.8361000000000
C32	1.4714000000000	3.8999000000000	2.0873000000000
C33	1.2404000000000	3.8853000000000	0.7112000000000
C34	0.6936000000000	2.7470000000000	0.1318000000000
H35	0.3196000000000	0.7748000000000	2.7330000000000
H36	1.2965000000000	2.7346000000000	3.9085000000000
H37	1.8987000000000	4.7747000000000	2.5669000000000
H38	1.4797000000000	4.7394000000000	0.0877000000000
H39	0.4999000000000	2.6833000000000	−0.9317000000000
C40	−2.4775000000000	−2.0362000000000	−1.3476000000000
C41	−2.9270000000000	−3.1436000000000	−2.0575000000000
C42	−1.9976000000000	−3.9748000000000	−2.6800000000000
C43	−0.6428000000000	−3.6555000000000	−2.5719000000000
C44	−0.2680000000000	−2.5262000000000	−1.8541000000000
H45	−3.1899000000000	−1.3784000000000	−0.8753000000000
H46	−3.9953000000000	−3.3211000000000	−2.1045000000000
H47	−2.3170000000000	−4.8493000000000	−3.2380000000000
H48	0.1225000000000	−4.2668000000000	−3.0373000000000
H49	0.7721000000000	−2.2431000000000	−1.7608000000000
N50	−1.1692000000000	−1.7231000000000	−1.2457000000000
N51	0.3799000000000	1.6539000000000	0.8570000000000
P52	−3.6808000000000	1.1761000000000	0.7097000000000
P53	6.7541000000000	0.4026000000000	−0.5458000000000
O54	−3.1228000000000	2.4563000000000	1.2757000000000
O55	6.9318000000000	1.3323000000000	0.6354000000000
O56	7.0248000000000	−1.1710000000000	−0.0693000000000
O57	−5.1736000000000	0.9569000000000	0.5538000000000
O58	7.5281000000000	0.6118000000000	−1.8257000000000
O59	0.9144000000000	0.2186000000000	−1.6087000000000
H60	6.3664000000000	−1.3923000000000	0.6126000000000
O61	−5.4726000000000	2.6940000000000	−1.7603000000000
H62	−4.6118000000000	3.1584000000000	−1.6194000000000
O63	−2.9532000000000	3.8580000000000	−1.1387000000000

H64	-3.0191000000000	3.6234000000000	-0.1849000000000
H65	-2.3632000000000	3.1437000000000	-1.4455000000000
O66	-4.6300000000000	0.4949000000000	-3.1908000000000
H67	-3.7969000000000	0.4302000000000	-2.6933000000000
H68	-5.0108000000000	1.3239000000000	-2.7992000000000
O69	-5.6060000000000	-1.2565000000000	-1.2043000000000
H70	-5.3575000000000	-0.7306000000000	-1.9977000000000
H71	-5.5673000000000	-0.5821000000000	-0.4963000000000
H72	-5.5644000000000	2.1501000000000	-0.9496000000000

S3.6 5. RuIV-OO - Triplet, Charge = 0

O1	-3.0890000000000	0.5817000000000	-1.0346000000000
O2	-1.6993000000000	0.9585000000000	-1.2263000000000
Ru3	-0.4478000000000	-0.0015000000000	-0.1057000000000
C4	2.9088000000000	-0.2957000000000	-0.1558000000000
C5	2.2537000000000	0.3862000000000	-1.2623000000000
C6	3.0905000000000	1.0241000000000	-2.2291000000000
C7	4.4517000000000	1.0185000000000	-2.0718000000000
C8	5.0063000000000	0.3580000000000	-0.9473000000000
H9	2.5983000000000	1.5110000000000	-3.0646000000000
H10	5.1127000000000	1.4944000000000	-2.7872000000000
C11	2.2341000000000	-1.1066000000000	0.8970000000000
C12	3.0092000000000	-1.9679000000000	1.6893000000000
C13	2.3999000000000	-2.7213000000000	2.6838000000000
H14	4.0725000000000	-2.0269000000000	1.5053000000000
C15	0.2919000000000	-1.7351000000000	2.0984000000000
C16	1.0288000000000	-2.6006000000000	2.9039000000000
H17	2.9895000000000	-3.3966000000000	3.2940000000000
H18	0.5526000000000	-3.1732000000000	3.6887000000000
C19	-1.1572000000000	-1.4772000000000	2.2882000000000
C20	-1.9178000000000	-2.0635000000000	3.2995000000000
C21	-3.0162000000000	-0.2163000000000	1.5718000000000
C22	-3.2624000000000	-1.7189000000000	3.4320000000000
H23	-1.4759000000000	-2.7803000000000	3.9790000000000
C24	-3.8172000000000	-0.7816000000000	2.5655000000000
H25	-3.8645000000000	-2.1767000000000	4.2094000000000
H26	-4.8572000000000	-0.4864000000000	2.6362000000000
N27	0.8992000000000	-1.0277000000000	1.1037000000000
N28	-1.7257000000000	-0.5800000000000	1.4339000000000
N29	4.2363000000000	-0.2731000000000	-0.0446000000000
C30	0.1948000000000	1.9115000000000	2.2086000000000
C31	0.4593000000000	3.1192000000000	2.8414000000000
C32	0.5450000000000	4.2805000000000	2.0735000000000
C33	0.3624000000000	4.1886000000000	0.6932000000000
C34	0.1015000000000	2.9477000000000	0.1285000000000
H35	0.1163000000000	0.9931000000000	2.7752000000000
H36	0.5911000000000	3.1381000000000	3.9172000000000
H37	0.7486000000000	5.2381000000000	2.5414000000000
H38	0.4166000000000	5.0627000000000	0.0544000000000
H39	-0.0560000000000	2.8306000000000	-0.9357000000000
C40	-2.1469000000000	-2.3220000000000	-1.1715000000000
C41	-2.4454000000000	-3.5166000000000	-1.8152000000000
C42	-1.4112000000000	-4.2715000000000	-2.3655000000000
C43	-0.1036000000000	-3.7930000000000	-2.2592000000000
C44	0.1219000000000	-2.5864000000000	-1.6115000000000
H45	-2.9464000000000	-1.7230000000000	-0.7602000000000
H46	-3.4829000000000	-3.8253000000000	-1.8710000000000
H47	-1.6155000000000	-5.2106000000000	-2.8697000000000
H48	0.7366000000000	-4.3381000000000	-2.6739000000000
H49	1.1225000000000	-2.1841000000000	-1.5239000000000

N50	-0.8817000000000	-1.8599000000000	-1.0704000000000
N51	0.0222000000000	1.8257000000000	0.8742000000000
P52	-3.7020000000000	1.0805000000000	0.4589000000000
P53	6.8132000000000	0.4036000000000	-0.6166000000000
O54	-3.1232000000000	2.4189000000000	0.8262000000000
O55	6.9960000000000	1.3814000000000	0.5234000000000
O56	7.0803000000000	-1.1399000000000	-0.0900000000000
O57	-5.1847000000000	0.8264000000000	0.3115000000000
O58	7.5230000000000	0.5594000000000	-1.9346000000000
O59	0.9779000000000	0.4620000000000	-1.4608000000000
H60	6.7050000000000	-1.2485000000000	0.8002000000000
O61	-5.5303000000000	2.1424000000000	-2.2406000000000
H62	-4.7077000000000	2.6816000000000	-2.1556000000000
O63	-3.0774000000000	3.5075000000000	-1.7695000000000
H64	-3.0760000000000	3.3643000000000	-0.7960000000000
H65	-2.4767000000000	2.8020000000000	-2.0663000000000
O66	-4.6107000000000	-0.2051000000000	-3.3497000000000
H67	-3.7125000000000	-0.1582000000000	-2.9844000000000
H68	-4.9942000000000	0.6585000000000	-3.0424000000000
O69	-5.2928000000000	-1.7086000000000	-1.0650000000000
H70	-5.1252000000000	-1.2758000000000	-1.9338000000000
H71	-5.4147000000000	-0.9384000000000	-0.4770000000000
H72	-5.6230000000000	1.7404000000000	-1.3518000000000

S3.7 6. RuIV-OO + OH- - Triplet, Charge = -1

O1	2.8821000000000	0.6823000000000	-1.7487000000000
O2	1.5432000000000	0.0514000000000	-1.7981000000000
Ru3	0.2204000000000	0.7079000000000	-0.5056000000000
C4	-3.0320000000000	0.0872000000000	-0.0470000000000
C5	-2.4587000000000	-0.1945000000000	-1.3282000000000
C6	-3.2432000000000	-0.9756000000000	-2.2167000000000
C7	-4.4714000000000	-1.4633000000000	-1.8289000000000
C8	-4.9464000000000	-1.1686000000000	-0.5330000000000
H9	-2.8307000000000	-1.1741000000000	-3.2017000000000
H10	-5.0789000000000	-2.0604000000000	-2.5017000000000
C11	-2.4372000000000	0.9733000000000	0.9765000000000
C12	-3.2654000000000	1.5404000000000	1.9678000000000
C13	-2.7242000000000	2.3600000000000	2.9417000000000
H14	-4.3235000000000	1.3228000000000	1.9395000000000
C15	-0.5577000000000	2.0328000000000	1.9684000000000
C16	-1.3472000000000	2.6037000000000	2.9572000000000
H17	-3.3620000000000	2.8060000000000	3.6975000000000
H18	-0.9120000000000	3.2203000000000	3.7328000000000
C19	0.9159000000000	2.1384000000000	1.9375000000000
C20	1.6663000000000	2.8420000000000	2.8851000000000
C21	2.8853000000000	1.3814000000000	0.8939000000000
C22	3.0552000000000	2.8188000000000	2.8139000000000
H23	1.1739000000000	3.4009000000000	3.6705000000000
C24	3.6742000000000	2.0664000000000	1.8157000000000
H25	3.6461000000000	3.3700000000000	3.5376000000000
H26	4.7528000000000	2.0007000000000	1.7356000000000
N27	-1.1111000000000	1.2571000000000	0.9850000000000
N28	1.5367000000000	1.4468000000000	0.9419000000000
N29	-4.2423000000000	-0.4097000000000	0.3028000000000
C30	0.4949000000000	-1.5634000000000	1.5218000000000
C31	0.6760000000000	-2.8657000000000	1.9707000000000
C32	0.5568000000000	-3.9159000000000	1.0600000000000
C33	0.2371000000000	-3.6211000000000	-0.2654000000000
C34	0.0719000000000	-2.2941000000000	-0.6403000000000
H35	0.5988000000000	-0.7196000000000	2.1921000000000

H36	0.9214000000000	-3.0425000000000	3.0118000000000
H37	0.7198000000000	-4.9414000000000	1.3733000000000
H38	0.1376000000000	-4.4019000000000	-1.0103000000000
H39	-0.1551000000000	-2.0111000000000	-1.6603000000000
C40	1.2745000000000	3.3881000000000	-1.5901000000000
C41	1.2318000000000	4.6861000000000	-2.0866000000000
C42	-0.0064000000000	5.2902000000000	-2.3035000000000
C43	-1.1635000000000	4.5635000000000	-2.0213000000000
C44	-1.0443000000000	3.2689000000000	-1.5310000000000
H45	2.2124000000000	2.8762000000000	-1.4237000000000
H46	2.1602000000000	5.2055000000000	-2.2967000000000
H47	-0.0689000000000	6.3037000000000	-2.6866000000000
H48	-2.1502000000000	4.9854000000000	-2.1765000000000
H49	-1.9183000000000	2.6710000000000	-1.3078000000000
N50	0.1552000000000	2.6888000000000	-1.3107000000000
N51	0.2057000000000	-1.2810000000000	0.2373000000000
P52	3.6522000000000	0.2593000000000	-0.3414000000000
P53	-6.5210000000000	-1.8755000000000	0.1038000000000
O54	3.2362000000000	-1.1495000000000	0.0574000000000
O55	-6.1919000000000	-3.0854000000000	0.9527000000000
O56	-7.0148000000000	-0.6175000000000	1.0780000000000
O57	5.1058000000000	0.6218000000000	-0.5001000000000
O58	-7.5112000000000	-1.9768000000000	-1.0328000000000
O59	-1.2980000000000	0.2401000000000	-1.7714000000000
H60	-6.2357000000000	-0.3207000000000	1.5824000000000
O61	5.6668000000000	-3.6874000000000	-1.3154000000000
H62	5.1748000000000	-4.2538000000000	-0.6816000000000
O63	3.5648000000000	-4.8659000000000	0.2050000000000
H64	3.6040000000000	-4.2689000000000	0.9789000000000
H65	3.1370000000000	-4.3166000000000	-0.4975000000000
O66	3.0177000000000	-3.1105000000000	-1.8780000000000
H67	2.8921000000000	-2.3305000000000	-1.2943000000000
H68	4.0025000000000	-3.1910000000000	-1.9084000000000
O69	6.5446000000000	-1.6393000000000	0.3651000000000
H70	6.1125000000000	-0.8174000000000	0.0340000000000
H71	6.0078000000000	-1.8849000000000	1.1439000000000
H72	6.0022000000000	-2.9400000000000	-0.7617000000000
O73	4.3534000000000	-2.7276000000000	1.8077000000000
H74	3.8607000000000	-2.0681000000000	1.2127000000000

S3.8 7. OH-attack-TS - Triplet, Charge = -1

O1	4.8320000000000	-1.2778000000000	1.5032000000000
P2	3.5617000000000	-0.4607000000000	-0.7892000000000
O3	2.6266000000000	0.2995000000000	-2.1466000000000
O4	1.2563000000000	0.0734000000000	-2.1953000000000
Ru5	0.1737000000000	0.3838000000000	-0.5816000000000
C6	-3.0964000000000	0.1578000000000	0.0173000000000
C7	-2.6223000000000	-0.0496000000000	-1.3235000000000
C8	-3.5551000000000	-0.5284000000000	-2.2777000000000
C9	-4.8457000000000	-0.8252000000000	-1.8990000000000
C10	-5.2203000000000	-0.6317000000000	-0.5534000000000
H11	-3.2046000000000	-0.6595000000000	-3.2966000000000
H12	-5.5776000000000	-1.1952000000000	-2.6083000000000
C13	-2.3195000000000	0.7689000000000	1.1294000000000
C14	-2.9875000000000	1.2136000000000	2.2820000000000
C15	-2.2644000000000	1.7857000000000	3.3189000000000
H16	-4.0607000000000	1.0979000000000	2.3345000000000
C17	-0.2460000000000	1.4472000000000	2.0666000000000
C18	-0.8773000000000	1.9020000000000	3.2207000000000
H19	-2.7747000000000	2.1358000000000	4.2097000000000

H20	-0.3057000000000	2.3247000000000	4.0366000000000
C21	1.2228000000000	1.4609000000000	1.8755000000000
C22	2.1039000000000	2.1135000000000	2.7309000000000
C23	3.0181000000000	0.6551000000000	0.5733000000000
C24	3.4738000000000	2.0669000000000	2.4613000000000
H25	1.7369000000000	2.6710000000000	3.5831000000000
C26	3.9400000000000	1.3185000000000	1.3887000000000
H27	4.1687000000000	2.5968000000000	3.1042000000000
H28	4.9973000000000	1.2061000000000	1.1962000000000
N29	-0.9758000000000	0.9050000000000	1.0487000000000
N30	1.6960000000000	0.7876000000000	0.7838000000000
N31	-4.3626000000000	-0.1414000000000	0.3476000000000
C32	-0.2840000000000	-2.0982000000000	1.1499000000000
C33	-0.4869000000000	-3.4434000000000	1.4321000000000
C34	-0.4366000000000	-4.3692000000000	0.3918000000000
C35	-0.1740000000000	-3.9140000000000	-0.9019000000000
C36	0.0264000000000	-2.5579000000000	-1.1114000000000
H37	-0.2959000000000	-1.3567000000000	1.9347000000000
H38	-0.6765000000000	-3.7469000000000	2.4551000000000
H39	-0.5941000000000	-5.4256000000000	0.5842000000000
H40	-0.1193000000000	-4.5960000000000	-1.7427000000000
H41	0.2437000000000	-2.1579000000000	-2.0928000000000
C42	1.4209000000000	3.1031000000000	-1.3319000000000
C43	1.4851000000000	4.4596000000000	-1.6269000000000
C44	0.3090000000000	5.2090000000000	-1.6345000000000
C45	-0.8975000000000	4.5666000000000	-1.3542000000000
C46	-0.8861000000000	3.2080000000000	-1.0679000000000
H47	2.3085000000000	2.4855000000000	-1.3324000000000
H48	2.4467000000000	4.9103000000000	-1.8451000000000
H49	0.3310000000000	6.2709000000000	-1.8571000000000
H50	-1.8401000000000	5.1020000000000	-1.3545000000000
H51	-1.8039000000000	2.6772000000000	-0.8517000000000
N52	0.2549000000000	2.4866000000000	-1.0495000000000
N53	-0.0345000000000	-1.6613000000000	-0.1016000000000
P54	-6.8969000000000	-1.0957000000000	0.0524000000000
O55	2.9487000000000	-1.8327000000000	-0.7073000000000
O56	-6.7371000000000	-2.2924000000000	0.9618000000000
O57	-7.2720000000000	0.2494000000000	0.9524000000000
O58	4.9254000000000	-0.0993000000000	-1.3135000000000
O59	-7.8332000000000	-1.1076000000000	-1.1293000000000
O60	-1.4023000000000	0.1830000000000	-1.7668000000000
H61	-6.6745000000000	0.2907000000000	1.7183000000000
H62	4.9470000000000	-2.1648000000000	1.1169000000000
O63	2.4366000000000	-1.9539000000000	2.2704000000000
H64	3.3831000000000	-1.6184000000000	2.1322000000000
H65	2.2345000000000	-2.1937000000000	1.3525000000000
O66	7.0326000000000	-0.1450000000000	0.6399000000000
H67	6.5884000000000	0.2631000000000	-0.1253000000000
H68	6.2138000000000	-0.5996000000000	1.0729000000000
O69	6.8638000000000	-2.1498000000000	-1.2897000000000
H70	6.1560000000000	-1.5607000000000	-1.6242000000000
H71	7.1512000000000	-1.6178000000000	-0.5086000000000
O72	4.9150000000000	-3.8052000000000	-0.1476000000000
H73	4.1492000000000	-3.2410000000000	-0.3805000000000
H74	5.6677000000000	-3.3206000000000	-0.5621000000000

S3.9 8. RuIV-OO - Triplet , Charge = -1

O1	4.4401000000000	-1.6961000000000	1.2889000000000
P2	3.6796000000000	-1.0503000000000	-0.0442000000000
O3	2.3544000000000	0.1737000000000	-2.4790000000000

O4	1.0615000000000	-0.0485000000000	-2.4469000000000
Ru5	0.2133000000000	0.4822000000000	-0.6601000000000
C6	-3.0371000000000	0.4509000000000	-0.0012000000000
C7	-2.5951000000000	0.2945000000000	-1.3450000000000
C8	-3.5034000000000	-0.2761000000000	-2.2612000000000
C9	-4.7485000000000	-0.6938000000000	-1.8292000000000
C10	-5.0962000000000	-0.5295000000000	-0.4749000000000
H11	-3.1870000000000	-0.3806000000000	-3.2948000000000
H12	-5.4629000000000	-1.1389000000000	-2.5138000000000
C13	-2.2474000000000	1.1444000000000	1.0409000000000
C14	-2.9078000000000	1.7754000000000	2.1093000000000
C15	-2.1725000000000	2.4351000000000	3.0815000000000
H16	-3.9881000000000	1.7341000000000	2.1450000000000
C17	-0.1614000000000	1.8136000000000	1.9278000000000
C18	-0.7788000000000	2.4522000000000	2.9995000000000
H19	-2.6736000000000	2.9295000000000	3.9070000000000
H20	-0.1941000000000	2.9331000000000	3.7727000000000
C21	1.3043000000000	1.6753000000000	1.7948000000000
C22	2.1984000000000	2.3956000000000	2.5884000000000
C23	3.0548000000000	0.4477000000000	0.8406000000000
C24	3.5637000000000	2.1611000000000	2.4627000000000
H25	1.8359000000000	3.1414000000000	3.2841000000000
C26	3.9907000000000	1.1515000000000	1.6079000000000
H27	4.2757000000000	2.7338000000000	3.0474000000000
H28	5.0398000000000	0.8873000000000	1.5353000000000
N29	-0.8956000000000	1.1829000000000	0.9718000000000
N30	1.7422000000000	0.7661000000000	0.8736000000000
N31	-4.2593000000000	0.0439000000000	0.3943000000000
C32	-0.6615000000000	-1.9450000000000	0.9724000000000
C33	-1.1421000000000	-3.2271000000000	1.2131000000000
C34	-1.3540000000000	-4.0840000000000	0.1345000000000
C35	-1.0639000000000	-3.6266000000000	-1.1522000000000
C36	-0.5834000000000	-2.3341000000000	-1.3153000000000
H37	-0.4681000000000	-1.2570000000000	1.7832000000000
H38	-1.3368000000000	-3.5383000000000	2.2329000000000
H39	-1.7324000000000	-5.0891000000000	0.2921000000000
H40	-1.2069000000000	-4.2570000000000	-2.0229000000000
H41	-0.3397000000000	-1.9334000000000	-2.2915000000000
C42	1.9794000000000	2.9091000000000	-1.3814000000000
C43	2.3119000000000	4.2312000000000	-1.6565000000000
C44	1.3003000000000	5.1900000000000	-1.7033000000000
C45	-0.0160000000000	4.7848000000000	-1.4792000000000
C46	-0.2724000000000	3.4457000000000	-1.2096000000000
H47	2.7257000000000	2.1259000000000	-1.3537000000000
H48	3.3500000000000	4.4942000000000	-1.8274000000000
H49	1.5314000000000	6.2300000000000	-1.9112000000000
H50	-0.8393000000000	5.4900000000000	-1.5086000000000
H51	-1.2795000000000	3.0887000000000	-1.0353000000000
N52	0.7082000000000	2.5200000000000	-1.1560000000000
N53	-0.3912000000000	-1.5026000000000	-0.2701000000000
P54	-6.6922000000000	-1.1491000000000	0.1982000000000
O55	2.5587000000000	-1.9942000000000	-0.4265000000000
O56	-6.4097000000000	-2.3833000000000	1.0271000000000
O57	-7.0902000000000	0.1232000000000	1.1977000000000
O58	4.7508000000000	-0.6354000000000	-1.0442000000000
O59	-7.7135000000000	-1.1718000000000	-0.9136000000000
O60	-1.4099000000000	0.6942000000000	-1.7947000000000
H61	-6.3296000000000	0.2920000000000	1.7815000000000
H62	4.5462000000000	-2.6791000000000	1.1006000000000
O63	1.8800000000000	-2.6770000000000	2.3031000000000
H64	2.7449000000000	-2.3003000000000	2.5339000000000

H65	1.8985000000000	-2.5951000000000	1.3283000000000
O66	7.0406000000000	-0.7271000000000	0.5663000000000
H67	6.3928000000000	-0.4198000000000	-0.1057000000000
H68	6.4266000000000	-1.1284000000000	1.2096000000000
O69	6.4247000000000	-2.8425000000000	-1.3016000000000
H70	5.7789000000000	-2.1336000000000	-1.5276000000000
H71	6.9418000000000	-2.3788000000000	-0.6088000000000
O72	4.5877000000000	-4.1452000000000	0.2574000000000
H73	3.7556000000000	-3.9250000000000	-0.2032000000000
H74	5.2966000000000	-3.8352000000000	-0.3693000000000

S3.10 9. OO-Release - Triplet, Charge = -1

O1	3.5710000000000	-1.7550000000000	0.6757000000000
P2	3.6324000000000	-0.3052000000000	-0.0430000000000
O3	0.1646000000000	0.9929000000000	-4.0512000000000
O4	1.3725000000000	1.0466000000000	-3.9428000000000
Ru5	0.5743000000000	0.2647000000000	0.0178000000000
C6	-2.7604000000000	0.0660000000000	0.0955000000000
C7	-2.0940000000000	-0.5636000000000	-1.0186000000000
C8	-2.9206000000000	-1.3181000000000	-1.8986000000000
C9	-4.2747000000000	-1.4397000000000	-1.6901000000000
C10	-4.8459000000000	-0.7947000000000	-0.5735000000000
H11	-2.4289000000000	-1.7901000000000	-2.7452000000000
H12	-4.9052000000000	-2.0064000000000	-2.3681000000000
C13	-2.1420000000000	0.9224000000000	1.1464000000000
C14	-2.9708000000000	1.6210000000000	2.0510000000000
C15	-2.4252000000000	2.4115000000000	3.0484000000000
H16	-4.0407000000000	1.5206000000000	1.9424000000000
C17	-0.2389000000000	1.8154000000000	2.2692000000000
C18	-1.0367000000000	2.5079000000000	3.1698000000000
H19	-3.0717000000000	2.9497000000000	3.7339000000000
H20	-0.5902000000000	3.1111000000000	3.9511000000000
C21	1.2383000000000	1.8008000000000	2.3343000000000
C22	2.0443000000000	2.4313000000000	3.2884000000000
C23	3.1567000000000	0.8300000000000	1.3254000000000
C24	3.4278000000000	2.2583000000000	3.2355000000000
H25	1.6034000000000	3.0471000000000	4.0635000000000
C26	3.9965000000000	1.4439000000000	2.2470000000000
H27	4.0598000000000	2.7492000000000	3.9681000000000
H28	5.0685000000000	1.2880000000000	2.1918000000000
N29	-0.7955000000000	1.0493000000000	1.2652000000000
N30	1.8214000000000	1.0370000000000	1.3725000000000
N31	-4.0989000000000	-0.0801000000000	0.2616000000000
C32	0.5960000000000	-1.6718000000000	2.3911000000000
C33	0.4976000000000	-2.8677000000000	3.0923000000000
C34	0.2517000000000	-4.0482000000000	2.3906000000000
C35	0.1101000000000	-3.9748000000000	1.0055000000000
C36	0.2192000000000	-2.7401000000000	0.3757000000000
H37	0.7898000000000	-0.7441000000000	2.9137000000000
H38	0.6143000000000	-2.8629000000000	4.1707000000000
H39	0.1729000000000	-4.9983000000000	2.9096000000000
H40	-0.0799000000000	-4.8597000000000	0.4077000000000
H41	0.1145000000000	-2.6437000000000	-0.6946000000000
C42	1.7119000000000	2.7526000000000	-1.3586000000000
C43	1.7291000000000	3.9388000000000	-2.0848000000000
C44	0.5248000000000	4.4703000000000	-2.5455000000000
C45	-0.6557000000000	3.7858000000000	-2.2562000000000
C46	-0.5915000000000	2.6058000000000	-1.5231000000000
H47	2.6277000000000	2.3019000000000	-1.0034000000000
H48	2.6768000000000	4.4276000000000	-2.2835000000000

H49	0.5066000000000	5.3933000000000	-3.1164000000000
H50	-1.6198000000000	4.1518000000000	-2.5926000000000
H51	-1.4881000000000	2.0477000000000	-1.2888000000000
N52	0.5724000000000	2.0889000000000	-1.0753000000000
N53	0.4627000000000	-1.5955000000000	1.0491000000000
P54	-6.6308000000000	-0.9517000000000	-0.1679000000000
O55	2.4739000000000	-0.2113000000000	-1.0682000000000
O56	-6.7859000000000	-1.8875000000000	1.0133000000000
O57	-6.9455000000000	0.6155000000000	0.3132000000000
O58	5.0231000000000	0.0174000000000	-0.5485000000000
O59	-7.4114000000000	-1.1856000000000	-1.4415000000000
O60	-0.8201000000000	-0.5018000000000	-1.3183000000000
H61	-6.2203000000000	0.8862000000000	0.9041000000000
H62	3.2059000000000	-2.4417000000000	0.0133000000000
O63	1.1420000000000	-1.8368000000000	-2.7984000000000
H64	0.2777000000000	-1.5142000000000	-2.4604000000000
H65	1.7511000000000	-1.1773000000000	-2.3986000000000
O66	6.4783000000000	-2.2238000000000	0.1519000000000
H67	6.1887000000000	-1.2944000000000	-0.0088000000000
H68	5.7302000000000	-2.5534000000000	0.6805000000000
O69	5.2345000000000	-2.4692000000000	-2.3055000000000
H70	5.0579000000000	-1.5111000000000	-2.2869000000000
H71	5.7801000000000	-2.5734000000000	-1.4859000000000
O72	2.8835000000000	-3.4122000000000	-1.2370000000000
H73	2.1555000000000	-2.9886000000000	-1.7505000000000
H74	3.7056000000000	-3.1796000000000	-1.7433000000000

S4 References

(1) Bochevarov, A. D.; Harder, E.; Hughes, T. F.; Greenwood, J. R.; Braden, D. A.; Philipp, D. M.; Friesner, R. A. Jaguar: A high-performance quantum chemistry software program with strengths in life and materials sciences. *Int. J. Quantum Chem.* **2013**, *113*, 2110-2142.

(2) Becke, A. Density-Functional Thermochemistry. III. The Role of Exact Exchange. *J. Chem. Phys.* **1993**, *98*, 5648-5652.

(3) Grimme, S.; Antony, J.; Ehrlich, S.; Krieg, H. A consistent and accurate ab initio parametrization of density functional dispersion correction (DFT-D) for the 94 elements H-Pu. *J. Chem. Phys.* **2010**, *132*, 154104-154109.

(4) Goerigk, L.; Grimme, S. A thorough benchmark of density functional methods for general main group thermochemistry, kinetics, and noncovalent interactions. *Phys. Chem. Chem. Phys.* **2011**, *13*, 6670-6688.

(5) Hay, P. J.; Wadt, W. R. Ab initio effective core potentials for molecular calculations. Potentials for K to Au including the outermost core orbitals. *J. Chem. Phys.* **1985**, *82*, 299-310.

(6) Krishnan, R.; Binkley, J. S.; Seeger, R.; Pople, J. A. Self-consistent molecular orbital methods. XX. A basis set for correlated wave functions. *J. Chem. Phys.* **1980**, *72* (1), 650–654.

(7) Peverati, R.; Truhlar, D.G. Improving the Accuracy of Hybrid Meta-GGA Density Functionals by Range Separation. *J. Phys. Chem. Lett.* **2011**, *2* (21), 2810–2817.

(8) Adamo, C.; Barone, V. Toward Reliable Density Functional Methods without Adjustable Parameters: The PBE0 Model. *J. Chem. Phys.* **1999**, *110* (13), 6158–6170.

(9) Mardirossian, N.; Head-Gordon, M. ωB97M-V: A combinatorially optimized, range-separated hybrid, meta-GGA density functional with VV10 nonlocal correlation. *J. Chem. Phys.* **2016**, *144* (21), AIP Publishing.

(10) Nataliia Vereshchuk; Matheu, R.; Jordi Benet-Buchholz; Pipelier, M.; Lebreton, J.; Dubreuil, D.; Tessier, A.; Gimbert-Suriñach, C.; Ertem, M. Z.; Antoni Llobet. Second Coordination Sphere Effects in an Evolved Ru Complex Based on Highly Adaptable Ligand Results in Rapid Water Oxidation Catalysis. *J. Am. Chem. Soc.* **2020**, *142* (11), 5068–5077.

(11) Iron, M. A.; Janes, T. Evaluating Transition Metal Barrier Heights with the Latest Density Functional Theory Exchange–Correlation Functionals: The MOBH35 Benchmark Database. *J. Phys. Chem. A* **2019**, *123* (17), 3761–3781.

(12) Santra, G.; Sylvetsky, N.; Martin, J. M. L. Minimally Empirical Double-Hybrid Functionals Trained against the GMTKN55 Database: RevDSD-PBEP86-D4, RevDOD-PBE-D4, and DOD-SCAN-D4. *J. Phys. Chem. A* **2019**, *123* (24), 5129–5143.
